# Biogenic *E. coli* EPS-capped Ag/ZrO_2_ nanocomposites (Ag/ZrO_2_ NCs): synthesis, characterization, and multitarget biomedical activity

**DOI:** 10.1039/d6ra04410g

**Published:** 2026-09-11

**Authors:** Ibrahim M. Ibrahim, Zahraa Falah Azeez, Hanadi A. Alahmadi, Elham Mohammed Khatrawi, Mohammed K. Alghamdi, Mona Othman I. Albureikan, Noura Daffa, Faisal Miqad K. Albaqami, Ahmed Eid Alharbi, Dareen Alyousfi, Dalal Alfawaz, Ahmed Ghareeb

**Affiliations:** a Department of Clinical Pharmacology, Faculty of Medicine, King Abdulaziz University Jeddah Saudi Arabia imibrahim1@kau.edu.sa mkhalghamdi@kau.edu.sa dalfawaz@kau.edu.sa; b College of Biotechnology, University of Al-Qadisiyah Al Diwaniyah Iraq zahraa.azeez@qu.edu.iq; c College of Nursing, Al-Rayan National Colleges Madinah Saudi Arabia ha.Alahmadi@amc.edu.sa; d Department of Basic Medical Sciences, College of Medicine, Taibah University Madinah 42353 Saudi Arabia ekhatrawi@taibahu.edu.sa; e Department of Biological Sciences, Faculty of Science, King Abdulaziz University Jeddah 21589 Saudi Arabia malboraikan@kau.edu.sa; f Department of Clinical Microbiology and Immunology, Faculty of Medicine, King Abdulaziz University Jeddah Saudi Arabia ndafaa@kau.edu.sa; g Biology Department, Faculty of Science, Islamic University of Madinah Madinah 42351 Saudi Arabia falbaqami@iu.edu.sa; h Department of Medical Laboratory, College of Applied Medical Sciences in Yanbu, Taibah University, Yanbu Governorate Saudi Arabia aeharbi@taibahu.edu.sa; i Department of Clinical Biochemistry, Faculty of Medicine, King Abdulaziz University Jeddah Saudi Arabia dalyousfi@kau.edu.sa; j Botany and Microbiology Department, Faculty of Science, Suez Canal University Ismailia 41522 Egypt aghareeb@science.suez.edu.eg

## Abstract

This study reports the biogenic synthesis of Ag/ZrO_2_ nanocomposites (NCs) using *Escherichia coli* exopolysaccharide (EPS03) as a reducing and capping agent, with full physicochemical characterization and biomedical evaluation. XRD resolved monoclinic ZrO_2_ (JCPDS 37-1484) and FCC Ag (JCPDS 04-0783) as independent crystalline phases with no intermetallic formation. Characteristic reflections at 28.2° and 31.5° confirmed ZrO_2_, while Ag peaks appeared at 38.1°, 44.3°, 64.4°, and 77.4°, with Scherrer crystallite sizes of 15.5 nm and 29.0 nm, respectively. FTIR confirmed retained EPS biomolecular groups at the particle surface through hydroxyl stretching at 3444.81 cm^−1^, amide I/II bands at 1507–1636 cm^−1^, and metal oxide fingerprint absorptions at 675 and 517 cm^−1^ for Ag–O and Zr–O–Zr, respectively. XPS confirmed metallic Ag alongside a Zr^4+^ oxidation state at binding energies of 181.5 and 183.9 eV, with O 1s deconvolution resolving Zr–O and surface-adsorbed water contributions at 529.9 and 531.6 eV. HRTEM resolved lattice fringes with a 0.283 nm interplanar spacing, assigned to the (111) plane of monoclinic ZrO_2_. DLS recorded a *Z*-average hydrodynamic diameter of 75.6 nm (PDI = 0.201) and a zeta potential of −33.54 mV, confirming colloidal stability. MTT screening of WI-38 fibroblasts maintained viability above 95% up to 125 µg mL^−1^, with a cytotoxic IC_50_ of 292.66 ± 1 µg mL^−1^. DPPH and ABTS assays yielded IC_50_ values of 7.14 ± 0.13 and 6.68 ± 0.12 µg mL^−1^, respectively, approaching those of ascorbic acid. COX-1 and COX-2 inhibition IC_50_ values were 7.43 ± 0.2 and 10.5 ± 0.1 µg mL^−1^ relative to celecoxib, while BSA denaturation inhibition recorded an IC_50_ of 2.46 ± 0.112 µg mL^−1^, closely approaching diclofenac sodium at 1.72 ± 0.022 µg mL^−1^. Antimicrobial screening by agar well diffusion showed inhibition zones of 34, 26, and 18 mm against *Bacillus subtilis*, *Staphylococcus aureus*, and MRSA, respectively, each exceeding those of gentamicin (29, 24, and 14 mm). Against *Candida albicans*, a zone of 30 mm was recorded against fluconazole at 29 mm. Among Gram-negative strains, the NCs matched gentamicin against *E. coli* at 27 mm and showed a marginal advantage against *Salmonella typhi* at 21 mm *vs.* 20 mm. Broth microdilution yielded the lowest MIC of 7.8 µg mL^−1^ against both *B. subtilis* and *C. albicans*, with MBC/MIC and MFC/MIC ratios of 2 across most strains and a tightest bactericidal ratio of 1 against *E. coli*, keeping every strain within the ≤4 bactericidal/fungicidal cutoff. These results demonstrate that *E. coli* EPS03-mediated synthesis yields a physicochemically stable Ag/ZrO_2_ NC system with a well-defined multifunctional biomedical profile across oxidative, inflammatory, and microbial targets.

## Abstract 2

This study reports the biogenic synthesis of Ag/ZrO nanocomposites (NCs) using *Escherichia coli* exopolysaccharide (EPS03) as a reducing and capping agent, with full physicochemical characterization and biomedical evaluation.

## Graphical abstract

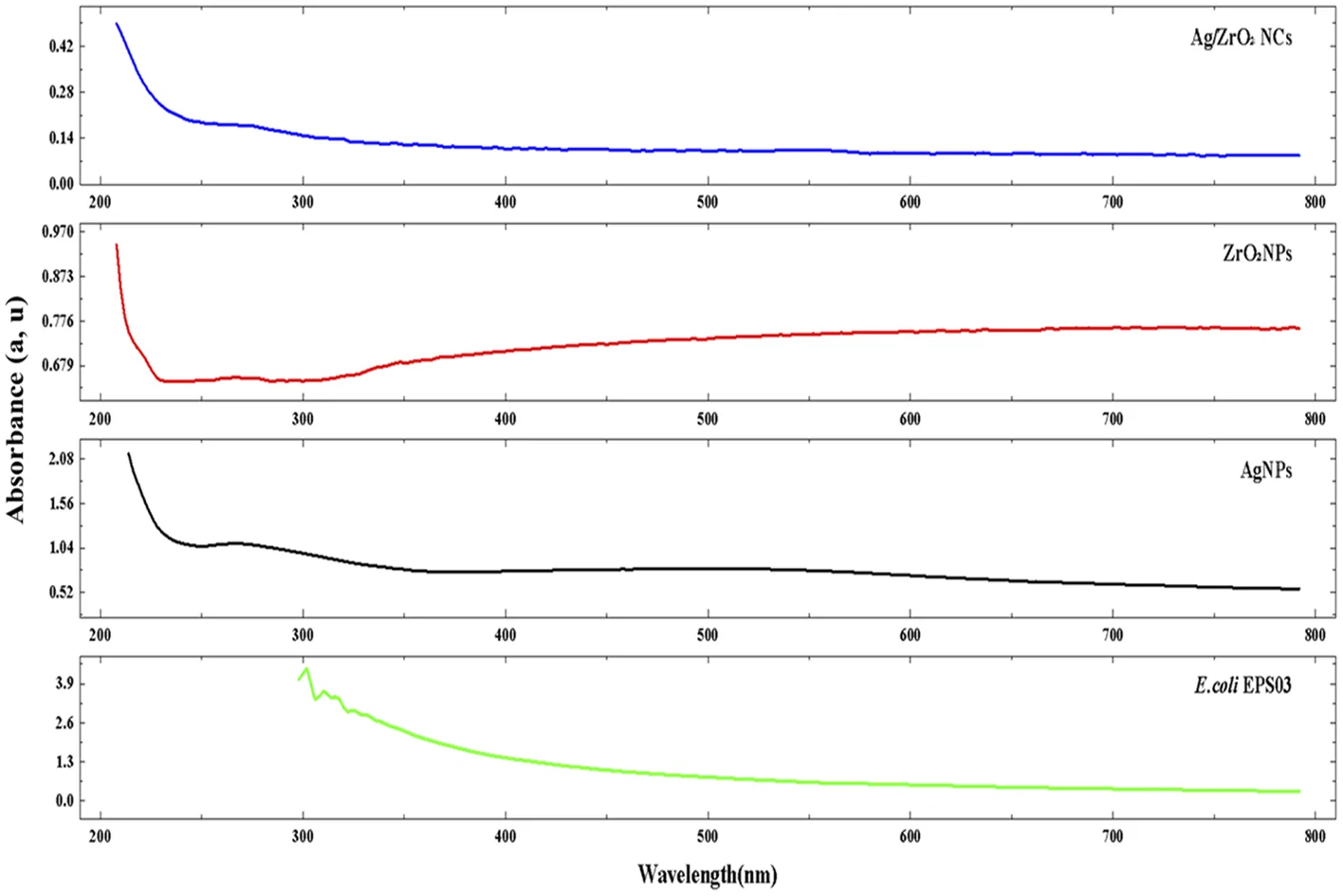

## Introduction

Infectious diseases, chronic inflammation, and oxidative stress collectively impose a substantial burden on global public health, compounded by the accelerating spread of antimicrobial resistance (AMR).^1^ Multidrug-resistant (MDR) pathogens, including methicillin-resistant *Staphylococcus aureus* (MRSA), extended-spectrum β-lactamase-producing *Klebsiella pneumoniae*, and drug-resistant *Candida* species, have rendered frontline antimicrobials progressively ineffective, prompting the World Health Organization to designate AMR among the gravest threats to human health.^2^ Beyond infection, dysregulated inflammatory responses and excessive reactive oxygen species (ROS) generation underlie a broad range of chronic conditions, from autoimmune and metabolic disorders to cardiovascular and neurological pathologies.^3^ Existing therapeutics addressing these overlapping conditions remain limited by drug toxicity, resistance development, and the lack of agents capable of simultaneously targeting microbial, oxidative, and inflammatory pathways.^4^ These gaps have directed research toward multifunctional nanomaterials as therapeutic platforms capable of acting across multiple biological targets within a single system.^5^

Metal oxide nanocomposites have gained considerable traction as multifunctional platforms owing to their tunable physicochemical properties and high surface-area-to-volume ratios.^6^ Unlike single-component nanoparticles, bimetallic composite architectures exploit synergistic interactions between constituents, generating antimicrobial, antioxidant, and anti-inflammatory activities that neither component produces independently at equivalent concentrations.^7^ Particle size, surface chemistry, and compositional ratios can be precisely modulated during synthesis to tailor activity profiles and stability, a degree of control largely unattainable with conventional small-molecule drugs.^8^ These attributes make metal oxide nanocomposites particularly suited to conditions where multiple pathological pathways require concurrent suppression and where monotherapy has proven inadequate.^9^

Silver nanoparticles (AgNPs) carry well-documented activity against a broad spectrum of Gram-positive and Gram-negative bacteria, fungi, and drug-resistant pathogens, operating through ROS generation, membrane disruption, and interference with intracellular enzymatic targets.^10^

Antioxidant and anti-inflammatory properties have also been recorded, linked to free radical scavenging and suppression of pro-inflammatory cytokine expression.^11^ Zirconium dioxide (ZrO_2_) nanoparticles, on the other hand, offer chemical stability, biocompatibility, and antioxidant radical scavenging capacity.^12^ While functioning as a structurally robust support matrix that anchors metal nanoparticles, limits aggregation, and preserves surface reactivity.^13^ The combination of the two components into a single nanocomposite is therefore compositionally justified, with Ag contributing direct biological activity against microbial, oxidative, and inflammatory targets. At the same time, ZrO_2_ provides structural reinforcement and supplementary redox functionality.^14^

Conventional chemical and physical nanoparticle synthesis routes depend on hazardous reducing agents and toxic stabilizers that limit biocompatibility and restrict biomedical applicability.^15^ Biogenic synthesis circumvents these drawbacks by exploiting biological systems as both reducing and capping platforms,^16^ producing nanoparticles that are surface-functionalized with biomolecules and are more compatible with biological environments.^17^ Among biological platforms, bacterial exopolysaccharides (EPS) are high molecular weight polymers composed of repeating sugar units decorated with uronic acids, acetyl groups, and peptide moieties.^18,19^ They carry hydroxyl, carboxyl, and amine functional groups directly involved in metal ion chelation and reduction, serving simultaneously as reducing and stabilizing matrices that prevent nanoparticle aggregation.^20^

*Escherichia coli*-derived EPS is particularly suited to this role; its well-characterized composition and abundance of metal-coordinating groups provide a reproducible scaffold for nanocomposite formation.^21^ The retained EPS corona on the NC surface may further contribute to biological activity, given that bacterial polysaccharides and glycoproteins independently exhibit antioxidant and immunomodulatory properties.^22^ Despite growing interest in biogenic metal oxide nanocomposites, Ag/ZrO_2_ NCs fabricated using bacterial EPS remain largely unreported, with no prior work addressing their synthesis *via E. coli* EPS or evaluating their biomedical potential. While Ag NPs and ZrO_2_ NPs have been studied individually.^23,24^ Their integration into a single EPS-capped nanocomposite, where constituent synergism and biomolecular surface functionalization may jointly amplify biological activity, has received little attention.^25^ Accordingly, this study reports the biogenic synthesis of Ag/ZrO_2_ NCs using *E. coli* EPS03, with physicochemical characterization conducted *via* UV-vis spectroscopy, FTIR, XRD, TEM, EDX, and zeta potential measurements, and systematic biomedical evaluation encompassing antioxidant (DPPH and ABTS), anti-inflammatory (COX inhibition and BSA denaturation), and antimicrobial activity against clinically relevant bacterial and fungal reference strains.

## Materials and methods

### Biogenic synthesis of *E. coli* EPS-capped Ag/ZrO_2_ NCs

*E. coli* EPS03 was dissolved in dH_2_O to a working concentration of 2 mg mL^−1^. The pH of both precursor solutions was adjusted to 7 with 0.1 N NaOH before EPS addition, and reactions were conducted at room temperature. Silver nitrate (AgNO_3_, 1 mM) was dissolved in 25 mL dH_2_O, combined with 25 mL of the EPS solution, and stirred at 600 rpm. In parallel, zirconyl nitrate (ZrO(NO_3_)_2_, 1 mM) was dissolved in 25 mL dH_2_O, combined with 25 mL of the EPS solution, and stirred under identical conditions.^26^ Both suspensions were maintained at 600 rpm for 48 h to allow EPS-guided metal ion reduction, nucleation, and surface capping. The two suspensions were then combined and agitated at 700 rpm for a further 24 h to permit composite phase integration.^27^ The suspension was then centrifuged at 10 000 rpm for 15 min. The supernatant was discarded, and the pellet was washed three times with deionized water, with centrifugation repeated between cycles, to remove unreacted Ag^+^ and Zr^4+^ ions. The recovered NCs were calcined and stored at 4 °C in sealed amber vials protected from light.

### Physicochemical characterization of biogenic Ag/ZrO_2_ NCs

Surface functional groups were characterized by FT-IR spectroscopy (Nicolet 6700, Thermo Fisher Scientific). Each sample was ground with KBr powder, pressed into a pellet, and spectra were recorded over the range 400–4000 cm^−1^. Absorption bands were assigned to identify functional groups present on the Ag/ZrO_2_ NCs surface.^28^ Thermal stability of EPS was assessed by thermogravimetric analysis using a TA Instruments SDT Q600 under a continuous nitrogen flow, with a sample mass of 7.77 mg heated from 25 °C to 600 °C at a rate of 15 °C min^−1^,^29^ and for metal biogenic NPs it was heated up to 1000 °C.^30^ Phase composition and crystallinity were examined by powder XRD on a PANalytical X'Pert Pro MRD diffractometer (CuKα; *λ* = 1.54 Å; 40 kV, 30 mA). Patterns were collected across a 2*θ* range of 10°–80° and used to index the crystal structure of the Ag/ZrO_2_ NCs.^31^ XPS was performed on a Thermo Scientific instrument (400 µm spot size, flood gun on), with spectra processed in Thermo Avantage software. High-resolution TEM and SAED were performed on the same JEOL JEM-2100 Plus instrument in high-resolution mode.^32^ Elemental composition was determined by EDX on a JEOL JSM-6360LA instrument, which confirmed Ag, Zr, and O as the constituent elements of the Ag/ZrO_2_ NCs.^33^ Hydrodynamic size and zeta potential of the Ag/ZrO_2_ NCs were measured on a Malvern Nano-ZS Zetasizer (Malvern Instruments Ltd, UK). NCs were dispersed in Milli-Q water before measurement; zeta potential was recorded sequentially under the same conditions to evaluate surface charge.^34^

### Biomedical evaluation of the biogenic Ag/ZrO_2_ NCs

#### Cytotoxicity evaluation of Ag/ZrO_2_ NCs on WI-38 normal cells (MTT assay)

WI-38 normal human lung fibroblast cells, purchased from VACSERA (Holding Company for Vaccines, Sera and Drugs), Cairo, Egypt, were plated at 1 × 10^5^ cells per ml (100 µl per well) in 96-well plates and left at 37 °C for 24 hours so a confluent monolayer could form. Wells were rinsed twice with wash media, then two-fold serial dilutions of Ag/ZrO_2_ NCs, prepared in RPMI-1640 (Lonza, USA) with 2% fetal bovine serum (FBS), 100 units per mL penicillin, and 100 µg mL^−1^ streptomycin, were dispensed (0.1 ml per well) in triplicate.^35^ Three wells received no treatment and served as controls. The plates were incubated at 37 °C and checked under the microscope for morphological signs of toxicity, such as rounding, shrinkage, granulation, and breakdown of the monolayer.^36^ MTT solution (5 mg mL^−1^ in PBS, 20 µl per well) was added next, shaken at 150 rpm for 5 minutes, and incubated at 37 °C in 5% CO_2_ for 4 hours. The medium was then removed, formazan crystals were dissolved in 200 µl DMSO with agitation, and absorbance was measured at 560 nm to determine cell viability against the untreated controls.^37^Cell viability (%) = (OD of treated cells/OD of untreated cells) × 100

#### Antioxidant activity

##### DPPH free radical scavenging assay

The DPPH radical scavenging capacity of the Ag/ZrO_2_ NCs was measured following the method of Khowdiary *et al.* (2024) with minor modifications. A stock solution of 2,2-diphenyl-1-picrylhydrazyl (DPPH) at 0.1 mM was prepared in ethanol. Serial dilutions of the Ag/ZrO_2_ NCs were prepared in ethanol across a concentration range of 1.9–1000 µg mL^−1^. For the assay, 3 mL of each NC suspension was mixed with 1 mL of the DPPH stock solution, briefly vortexed, and then kept in the dark at room temperature for 30 min. Ascorbic acid (AA) was run in parallel as a positive control under identical conditions. Absorbance of the reaction mixtures was read at 517 nm on a Milton Roy UV-vis spectrophotometer.^38^ The percentage radical scavenging activity was calculated as:DPPH scavenging% = [(AA_absorbance_ − Ag/ZrO_2_ NCs_absorbance_)/AA_absorbance_] × 100

##### ABTS˙^+^ radical cation scavenging assay

The ABTS˙^+^ radical cation stock was prepared by reacting 7 mM 2,2′-azino-bis(3-ethylbenzothiazoline-6-sulfonic acid) (ABTS) with 2.45 mM potassium persulfate (K_2_S_2_O_8_) and leaving the mixture undisturbed in the dark at room temperature for 12–16 h until a stable radical was formed.^35^ Sample aliquots of 0.07 mL of each Ag/ZrO_2_ NCs concentration were added to 3 mL of the working ABTS˙^+^ reagent, mixed, and left to react for 6 min at room temperature. Absorbance was then read at 734 nm on a UV-vis spectrophotometer, with AA run as the positive control under the same conditions.^37^ Inhibition of the ABTS˙^+^ radical was expressed as:ABTS˙^+^ inhibition% = [(AA_absorbance_ − Ag/ZrO_2_ NCs_absorbance_)/AA_absorbance_] × 100

#### Anti-inflammatory assessment of the biogenic Ag/ZrO_2_ NCs

##### COX enzyme inhibition assay

The capacity of Ag/ZrO_2_ NCs to inhibit COX-1 and COX-2 isoenzymes was evaluated using commercially available COX-1 (catalog no. k548) and COX-2 (catalog no. k547) inhibitor screening kits obtained from Biovision, USA. The prepared NCs were dissolved in DMSO and subjected to testing across a concentration gradient ranging from 0.5 to 1000 µg mL^−1^, with each reaction carried out in a total volume of 1 mL.^39^ Celecoxib served as the positive reference inhibitor for both isoenzymes throughout the assay.^40^ The percentage of COX inhibition was determined according to the following equation:COX-inhibition% = [(celecoxib_absorbance_ − Ag/ZrO_2_ NCs_absorbance_)/celecoxib_absorbance_] × 100

##### BSA protein denaturation assay

Each test NC (50 µL) was combined with 450 µL of 1% BSA aqueous solution, and eight working concentrations were prepared: 1.56, 3.12, 6.25, 12.5, 25, 50, 100, and 200 µg mL^−1^. The pH of each mixture was adjusted to 6.3 through dropwise addition of 1 N HCl. Samples were initially kept at room temperature for 20 minutes, then transferred to a water bath maintained at 55 °C for an additional 30 minutes. Following heat exposure, all mixtures were allowed to cool to room temperature, and absorbance readings were recorded at 670 nm using a Biosystem 310 Plus spectrophotometer, with diclofenac sodium serving as the reference standard.^41^ The inhibition percentage of BSA denaturation was calculated as follows:% BSA inhibition = [(diclofenac sodium_absorbance_ − Ag/ZrO_2_ NCs_absorbance_)/diclofenac sodium_absorbance_] × 100

#### Antimicrobial testing of biogenic Ag/ZrO_2_ NCs

The antimicrobial potential of Ag/ZrO_2_ NCs was assessed against ATCC reference strains *via* the agar well diffusion method.^42^ The Gram-positive bacterial panel consisted of *Bacillus subtilis* ATCC 6633, *Staphylococcus aureus* ATCC 6538, methicillin-resistant *Staphylococcus aureus* ATCC 33591 (MRSA), and *Enterococcus faecalis* ATCC 29212. Whereas the Gram-negative panel included *Escherichia coli* ATCC 8739, *Klebsiella pneumoniae* ATCC 13883, *Pseudomonas aeruginosa* ATCC 90274, *Salmonella typhi* ATCC 6539, and *Proteus vulgaris* NCTC 4175/ATCC 13315, all grown on Mueller–Hinton agar. Fungal strains, namely *Candida albicans* ATCC 10221, were tested on Sabouraud dextrose agar, with gentamicin and fluconazole used as antibacterial and antifungal reference standards, respectively.^22^ Inoculum suspensions prepared by broth microdilution were plated within 15 minutes, and three-directional streaking was applied to achieve uniform microbial distribution across the dried agar surface. Wells of 6 mm diameter were punched under aseptic conditions using sterile cork borers, and 100 µL of Ag/ZrO_2_ NCs at a concentration of 10 µg mL^−1^ in DMSO was loaded into each well.^43^

Bacterial plates were incubated at 37 °C for 24 hours, *Candida* sp. plates at 35 °C for 48 hours. Inhibition zones were recorded to the nearest millimeter at points where visible growth suppression was observed.^44^ Broth dilution assays were used to calculate MICs, MBCs, and MFCs independently.^42^

#### Statistical analysis

All experiments were performed in triplicate (*n* = 3), and results are expressed as mean ± standard deviation (SD). Data were processed using SPSS version 23. Normality of the distribution was assessed using the Shapiro–Wilk test. Comparisons between Ag/ZrO_2_ NCs and reference standards were carried out using an independent-samples *t*-test. IC_50_ values were calculated from the graph.

## Results and discussion

### Physicochemical characteristics of the biogenic Ag/ZrO_2_ NCs

EPS03 alone showed no distinct peak at 260 nm or 280 nm, ruling out nucleic acid or protein carryover and confirming a clean polysaccharide extract before use in synthesis. Applied to AgNP, the spectrum showed a shoulder near 266 nm and a broad surface plasmon resonance (SPR) band spanning roughly 450–520 nm, indicating the formation of metallic silver. ZrO_2_NP showed only the expected UV-edge rise below 230 nm due to its wide band gap, with a flat plateau afterward and no plasmonic feature, which is normal for a non-plasmonic oxide. The Ag/ZrO_2_ composite sat at the lowest overall, decaying smoothly with no bump in the 400–520 nm range, indicating that Ag's plasmon signal is damped upon pairing with ZrO_2_, suggesting interfacial coupling between the two phases rather than the components layered together (Fig. 1).

**Fig. 1:**
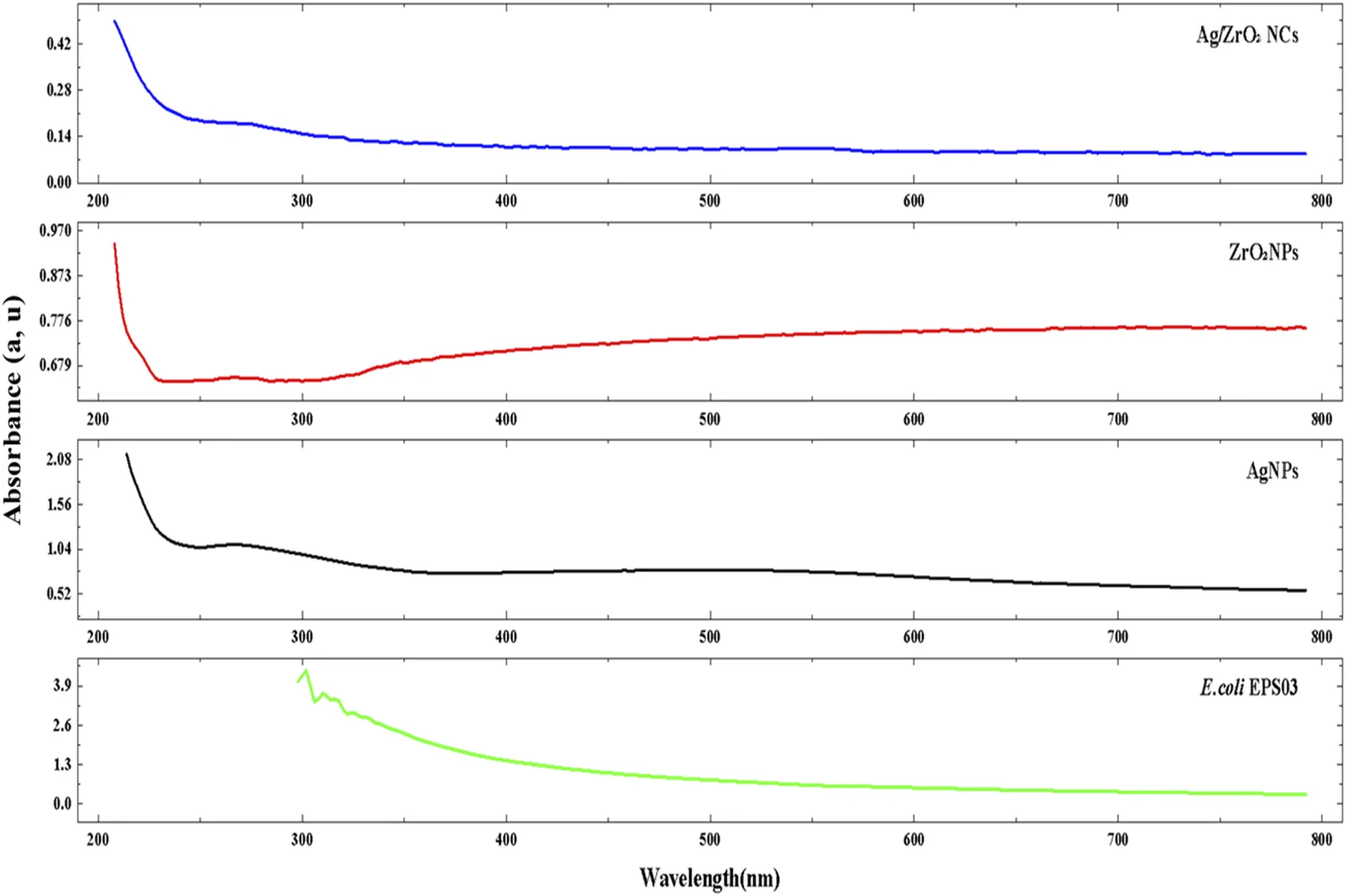
UV-vis spectra of *E. coli* EPS03, AgNP, ZrO_2_NP, and Ag/ZrO_2_ nanocomposites.

All three spectra carry the same bacterial EPS signature: an O–H stretch near 3400–3445 cm^−1^ and the amide I/II pair around 1500–1636 cm^−1^, confirming that the exopolysaccharide remains bound to the particle surface during synthesis. AgNPs and the composite share this capping layer almost peak for peak, while ZrO_2_NPs diverge below 900 cm^−1^, where Zr–OH and Zr–O–Zr modes take over. The composite spectrum keeps both metal fingerprints at once, Ag–O near 675 cm^−1^ and Zr–O–Zr near 517 cm^−1^, sitting as separate bands rather than merging into one the same phase-separation (Fig. 2) (Table 1).

**Fig. 2:**
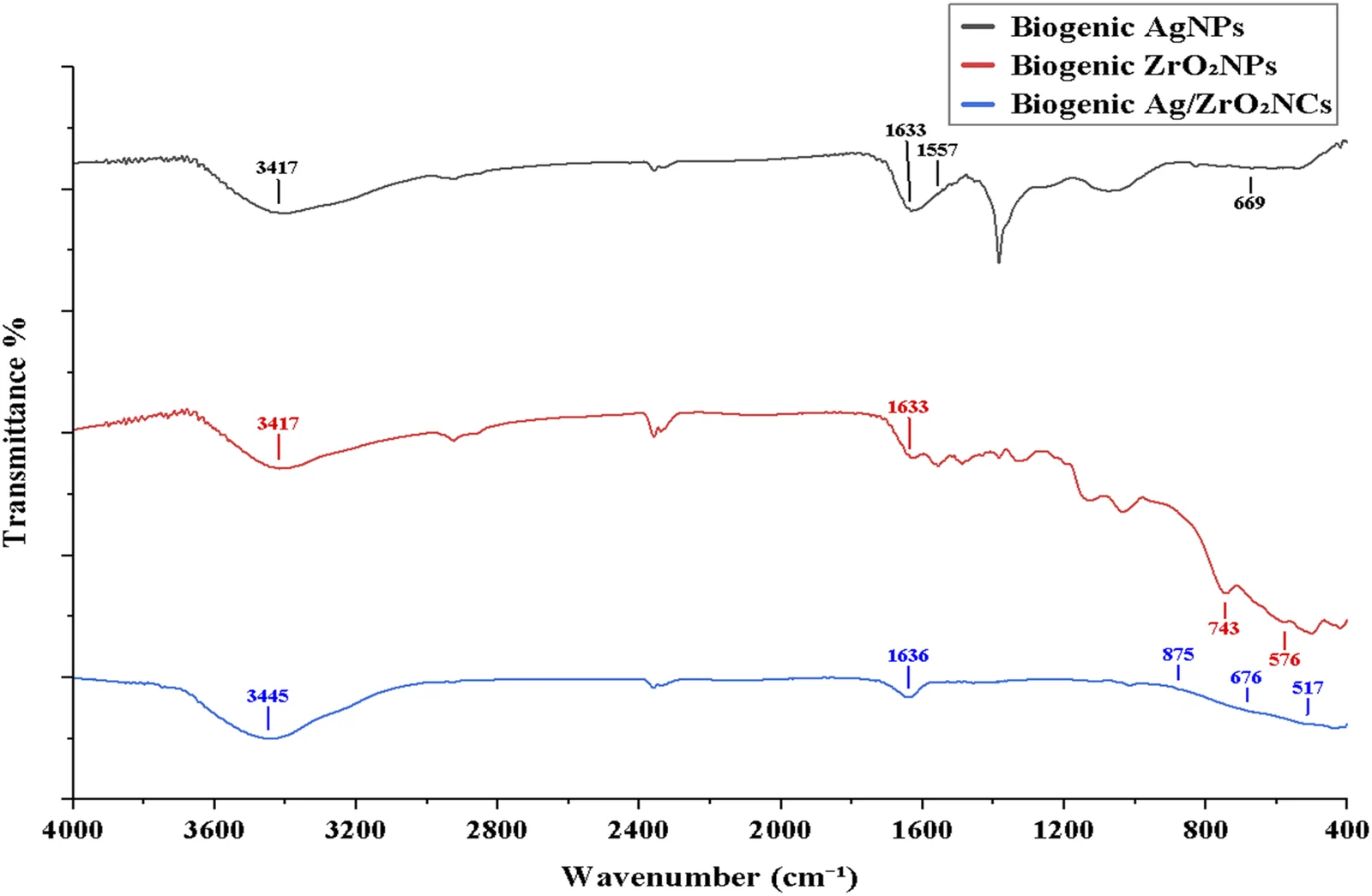
FTIR spectra of biogenic AgNPs, ZrO_2_NPs, and Ag/ZrO_2_NC, all synthesized using *E. coli* EPS03.

**Table 1:** FTIR band assignments for biogenic AgNPs, ZrO_2_NPs, and Ag/ZrO_2_NC, all synthesized using *E. coli* EPS03

Sample	Wavenumber (cm^−1^)	Assignment
AgNPs	3416.8	O–H stretch, EPS03 hydroxyls
AgNPs	2954.8/2925.5/2848.6	C–H stretch, EPS sugar backbone
AgNPs	1632.9	Amide I (C=O), bound EPS protein
AgNPs	1556.7/1540.2	Amide II (N–H/C–N)
AgNPs	1506.1/1453.9	C=C aromatic/C–H bend
AgNPs	1384.2	Carboxylate stretch
AgNPs	1254.9/1073.7	C–O–C, polysaccharide ring
AgNPs	896.6/830.5/761.3	C–H out-of-plane bend
AgNPs	668.8/620.0/543.1/459.3/414.8	Ag–O lattice modes
ZrO_2_NPs	3417.4	O–H stretch, surface hydroxyls
ZrO_2_NPs	2925.0/2853.5	C–H stretch, residual EPS
ZrO_2_NPs	1632.5	Amide I/adsorbed water
ZrO_2_NPs	1556.3	Amide II
ZrO_2_NPs	1487.4/1433.5/1384.2	Carboxylate/C–H bend
ZrO_2_NPs	1329.8/1194.8/1127.5/1033.1	C–O–C, EPS backbone
ZrO_2_NPs	742.9	Zr–OH bend
ZrO_2_NPs	576.0/500.4	Zr–O stretch
Ag/ZrO_2_NCs	3444.8	O–H stretch, EPS corona
Ag/ZrO_2_NCs	2923.5/2853.8	C–H stretch, retained EPS
Ag/ZrO_2_NCs	1636.4	Amide I (C=O)
Ag/ZrO_2_NCs	1557.3/1540.8/1520.5/1507.6	Amide II band cluster
Ag/ZrO_2_NCs	1456.3/1419.3	C–H bend/carboxylate
Ag/ZrO_2_NCs	1113.9/1015.3	C–O–C, EPS backbone
Ag/ZrO_2_NCs	874.9	Zr–OH bend
Ag/ZrO_2_NCs	675.5	Ag–O
Ag/ZrO_2_NCs	517.2	Zr–O–Zr

TG and DTG traces (Fig. 3) show four distinct thermal profiles. EPS03 loses 39.8% of its mass by 592 °C in three stages: moisture loss, amide cleavage, and polysaccharide breakdown, leaving 60.1% residue at the scan limit. The Ag/ZrO_2_ composite loses a similar 37.5% of its mass, almost all of it (24.0%) between 300 and 600 °C, settling near 62.1% before a small mass gain above 800 °C, consistent with partial oxidation of the composite's inorganic phase, bringing it to 62.5% residual mass at 987 °C. Free AgNP loses only 8.7% of its mass below 600 °C, but a further 28.3% up to 987 °C with no resolved DTG peak, ending at 63.0% residual mass.

**Fig. 3:**
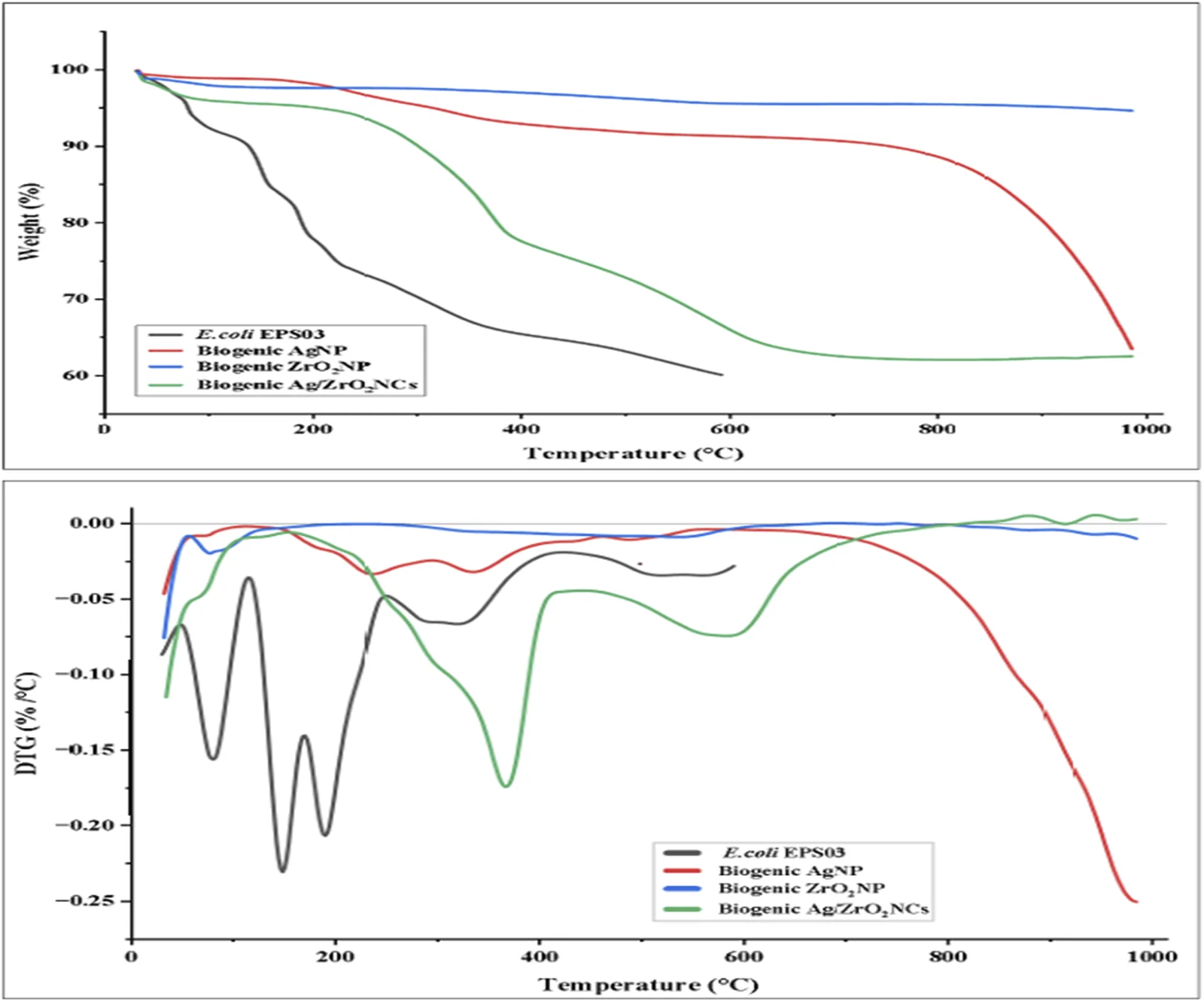
TG and DTG curves of EPS03, ZrO_2_NP, AgNP, and Ag/ZrO_2_ NC, showing mass-loss stages and residual mass at each scan limit (EPS03 to 600 °C; metal-containing samples to 1000 °C).

That loss falls to 3.5% once Ag is incorporated into the composite, supporting coupling between Ag and ZrO_2_. ZrO_2_NP is the most thermally stable sample, losing 5.4% of its mass and retaining 94.6% of its mass. Its EPS-related loss below 600 °C (4.4%) is roughly half that of AgNP (8.7%), indicating weaker binding of EPS to Zr–OH sites.

The XRD diffractogram resolved two crystalline phases throughout the full 2*θ* scan, monoclinic ZrO_2_ (*m*-ZrO_2_; JCPDS 37-1484) and face-centred cubic silver (FCC Ag; JCPDS 04-0783), free of any secondary phase lines. The (−111) reflection at 28.2° and the (111) at 31.5° appeared as two distinct, separated peaks, a splitting diagnostic of the monoclinic cell and absent in tetragonal ZrO_2_; Bragg-calculated *d*-spacings across all ZrO_2_ reflections at 24.1°, 28.2°, 31.5°, and 49.3° fell within 0.002 Å of JCPDS 37-1484. Four FCC silver reflections at 38.1°, 44.3°, 64.4°, and 77.4° matched JCPDS 04-0783 within 0.001 Å, with the (111) > (200) > (220) > (311) intensity drop ruling out any preferred crystallographic orientation (Fig. 4). Scherrer calculations from peak half-widths, which were noticeably broader for ZrO_2_ than for Ag across the diffractogram, yielded phase-averaged crystallite sizes of 15.5 nm and 29.0 nm for ZrO_2_ and Ag, respectively, indicating that the EPS matrix curtailed ZrO_2_ growth during synthesis.

**Fig. 4:**
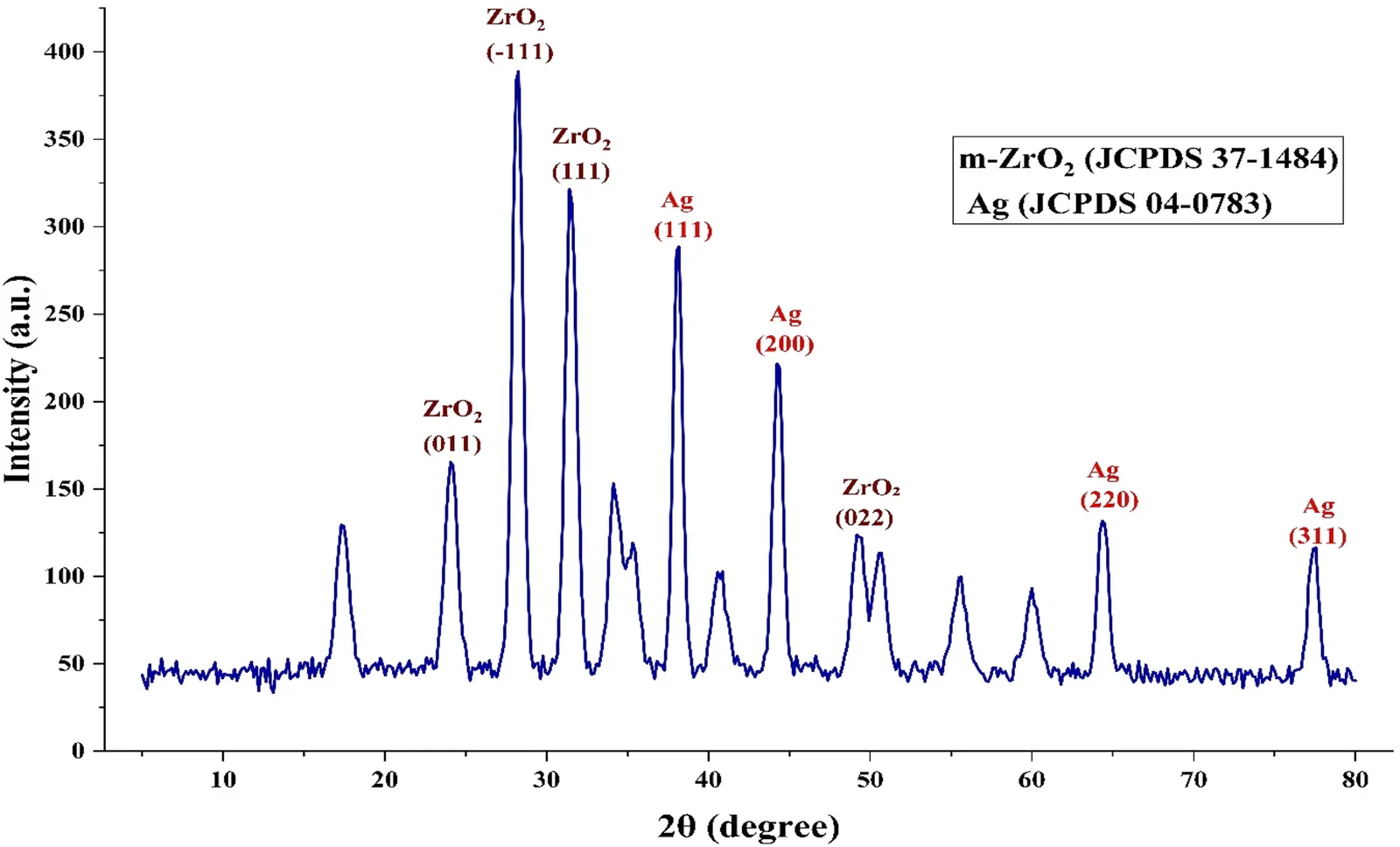
XRD pattern of biogenic *E. coli* EPS03-capped Ag/ZrO_2_ NCs.

To further confirm the structure of the nanocomposite and the oxidation states of the elements present, XPS analysis was performed, and the results are shown in Fig. 5.

**Fig. 5:**
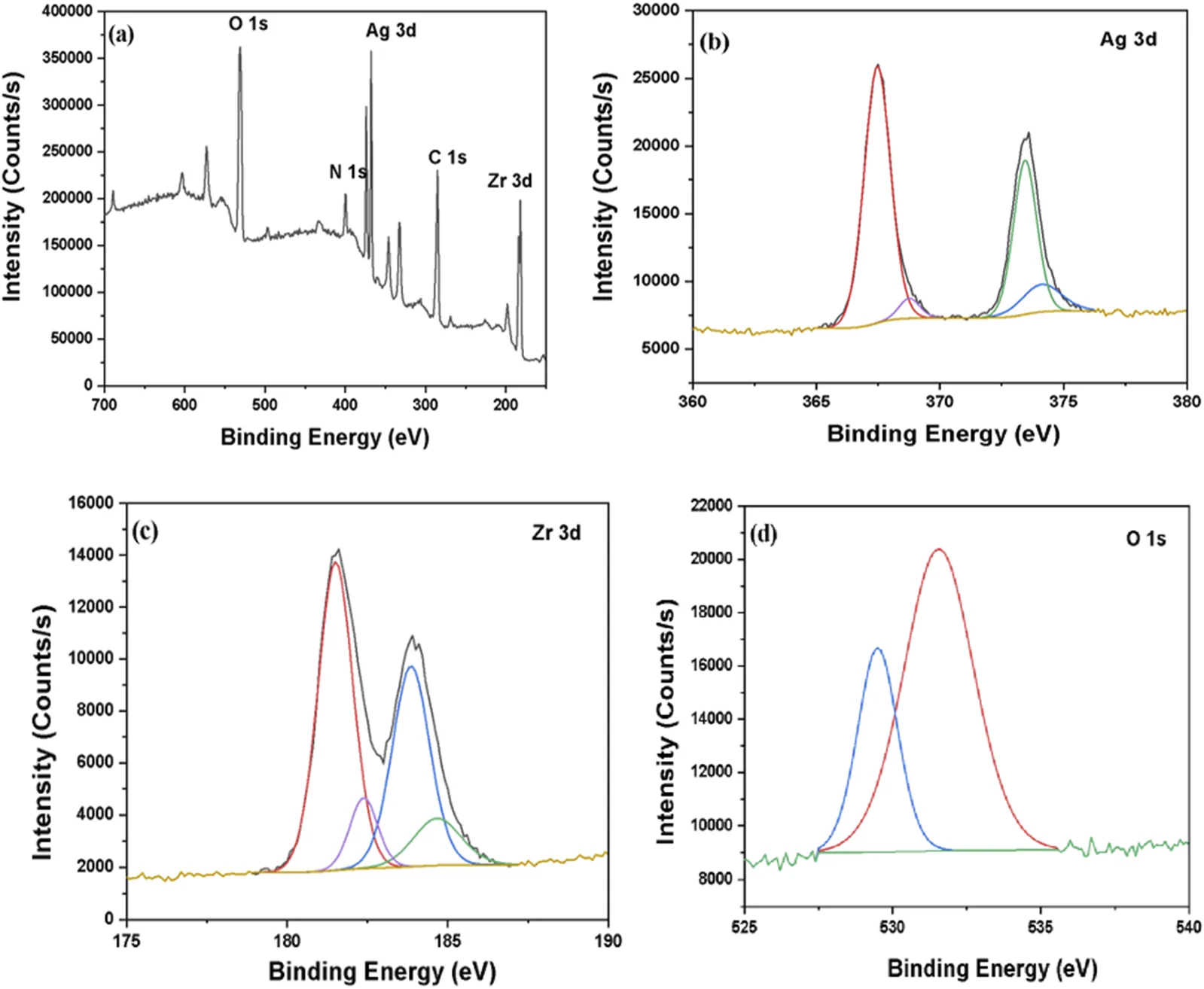
XPS spectra of Ag/ZrO_2_ (a) survey, (b) Ag 3d, (c) Zr 3d, and (d) O 1s.

Survey XPS (Fig. 5a) revealed peaks at binding energies of 182.5 eV, 285.2 eV, 399.8 eV, 367.8 eV, and 530.9 eV. These reflections corresponded to Zr 3d, C 1s, N 1s, Ag 3d, and O 1s, confirming the formation of Ag/ZrO_2_ nanocomposite. The presence of signals corresponding to C and N may be from the growth media, N, and C-containing species of bacteria, and may also be from the adsorbed CO_2_ on the composite surface.

The high-resolution XPS spectra of Ag 3d (Fig. 5b) show two main signals at 367.5 eV and 373.4 eV with spin–orbit splitting of 5.9 eV. These signals were attributed to Ag 3d_5/2_ and Ag 3d_3/2_ splitting of the metallic Ag^+^. Two other peaks were observed at 368.7 eV and 374 eV, which were attributed to metallic Ag.^45^ In comparison with the XRD pattern, the silver was clearly assigned to metallic Ag; however, XPS may detect a thin surface layer of Ag_2_O that is not visible by XRD. The Zr element's high-resolution XPS spectra (Fig. 5c) revealed two main distinct peaks with binding energies of 181.5 and 183.9 eV, attributed to Zr 3d_5/2_ and Zr 3d_3/2_ with a splitting of 2.4 eV, which are in good accord with the Zr^4+^ state.^46,47^ Here, two features at binding energies of 529.9 and 531.6 eV were resolved by deconvolution in the high-resolution O 1s XPS spectra (Fig. 5d). It was assumed that the metal–oxygen (Zr–O) bond was the source of the feature with lower-binding-energy electrons, and that surface-absorbed water molecules were the source of the feature with higher binding energy.^48^ These results confirmed the successful formation of Ag/ZrO_2_ nanocomposite.

The morphology and particle size of the prepared Ag/ZrO_2_ nanocomposite were further evaluated using TEM, HRTEM, and SAED (selected-area electron diffraction), and the results are presented in Fig. 6. In the TEM image (Fig. 6a), the material consists of irregularly shaped, closely aggregated nanoparticles distributed over a lighter, low-contrast region that can be attributed to the ZrO_2_ support. The relatively dark contrast of several particles is consistent with the presence of Ag nanoparticles, owing to silver's greater electron-scattering ability compared with zirconia. Although some agglomeration is visible, the Ag-containing particles appear to be dispersed throughout the composite rather than forming a separate continuous phase. The particle size distribution is not completely uniform, possibly due to nanoparticle growth and aggregation during synthesis. The average particle size estimated was 33–38 nm. The HRTEM image (Fig. 6b) shows well-resolved, parallel lattice fringes with an interplanar spacing of 0.283 nm. This value can be assigned to a crystalline plane of the ZrO_2_ phase, most plausibly the (111) plane of monoclinic ZrO_2_ (JCPDS No. 00-037-1484). However, definitive phase assignment should be made by comparison with the XRD data and the corresponding crystallographic database. The distinct lattice fringes confirm the high crystallinity of the imaged region and indicate the formation of an ordered oxide phase. The absence of a clearly resolved Ag lattice fringe in the selected area may be related to particle overlap, limited image contrast, or the orientation of the Ag nanoparticles.

**Fig. 6:**
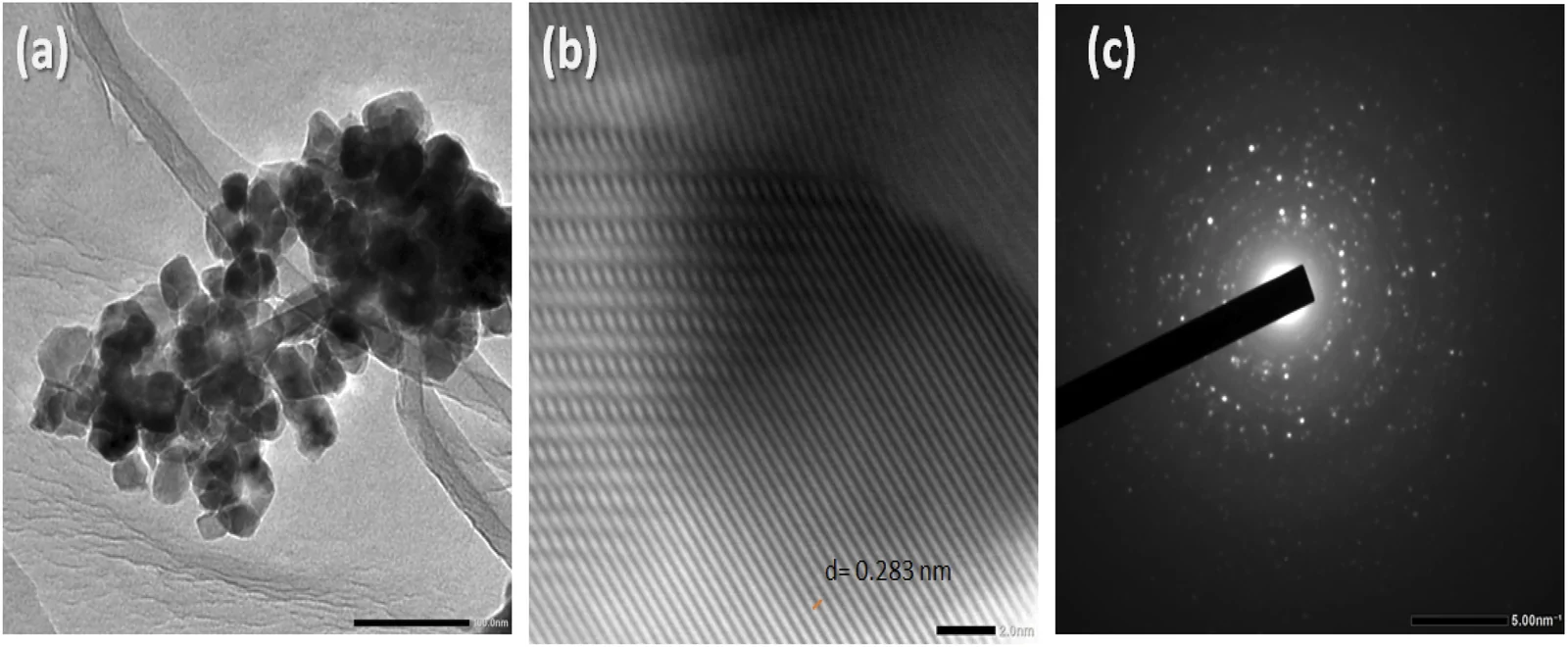
Electron microscopy analysis of Ag/ZrO_2_ NCs. (a) TEM overview, (b) HRTEM micrograph, and (c) SAED pattern.

The SAED pattern (Fig. 6c) exhibits a diffuse ring pattern accompanied by numerous discrete spots. This combination indicates that the sample is predominantly polycrystalline, with several nanocrystallites contributing to the diffraction pattern. At the same time, the partial continuity of the rings reflects the presence of randomly oriented crystallites.

The equivalent SAED pattern (Fig. 6c) exposes diffraction rings with interplanar spacing of 0.380, 0.295, 0.219, and 0.168 nm, due to (110), (111), (201), and (003) diffraction planes of *m*-ZrO_2_ (JCPDS No. 00-037-1484). Another interplanar spacing of 0.210 nm corresponded to (200) of the cubic Ag (JCPDS No. 00-004-0783). Overall, the TEM and HRTEM observations demonstrate the formation of crystalline, aggregated nanoparticles, whereas the SAED pattern confirms the polycrystalline nature of the Ag/ZrO_2_ nanocomposite.

EDX confirmed Ag and Zr as the constituent elements of the nanocomposite. Silver dominated the composition at 88.4 wt% (86.6 at%), with zirconium contributing 11.6 wt% (13.4 at%), yielding an Ag : Zr weight ratio of approximately 7.6 : 1 (Fig. 7).

**Fig. 7:**
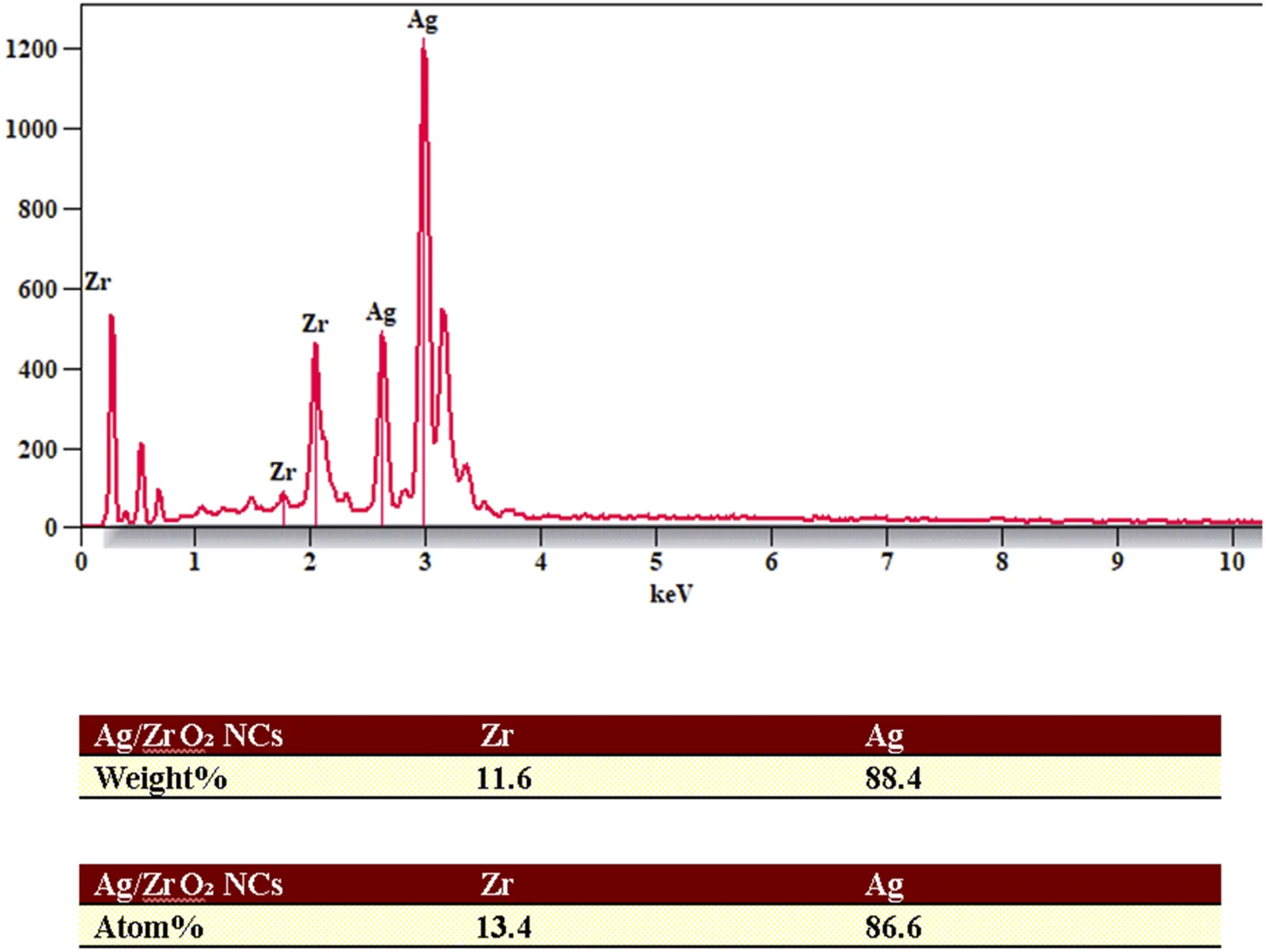
EDX spectrum of biogenic Ag/ZrO_2_ NCs, confirming Ag and Zr as the constituent elements with silver as the dominant inorganic phase.

DLS recorded a *Z*-average hydrodynamic diameter of 75.6 nm, a polydispersity index (PDI) of 0.201, and an autocorrelation intercept of 0.938, the latter confirming signal quality throughout the measurement. A single population dominated the intensity-weighted size distribution, centred at 75.6 nm and accounting for 96.8% of the total scattered intensity, with a width of 26.3 nm (Fig. 8). A minor population at 425 nm contributed the remaining 3.2%, consistent with a small aggregate fraction. The hydrodynamic diameter sits above the Scherrer-derived crystallite sizes of 15.5 nm for ZrO_2_ and 29.0 nm for Ag, the difference reflecting the EPS hydration shell surrounding the inorganic crystalline cores.

**Fig. 8:**
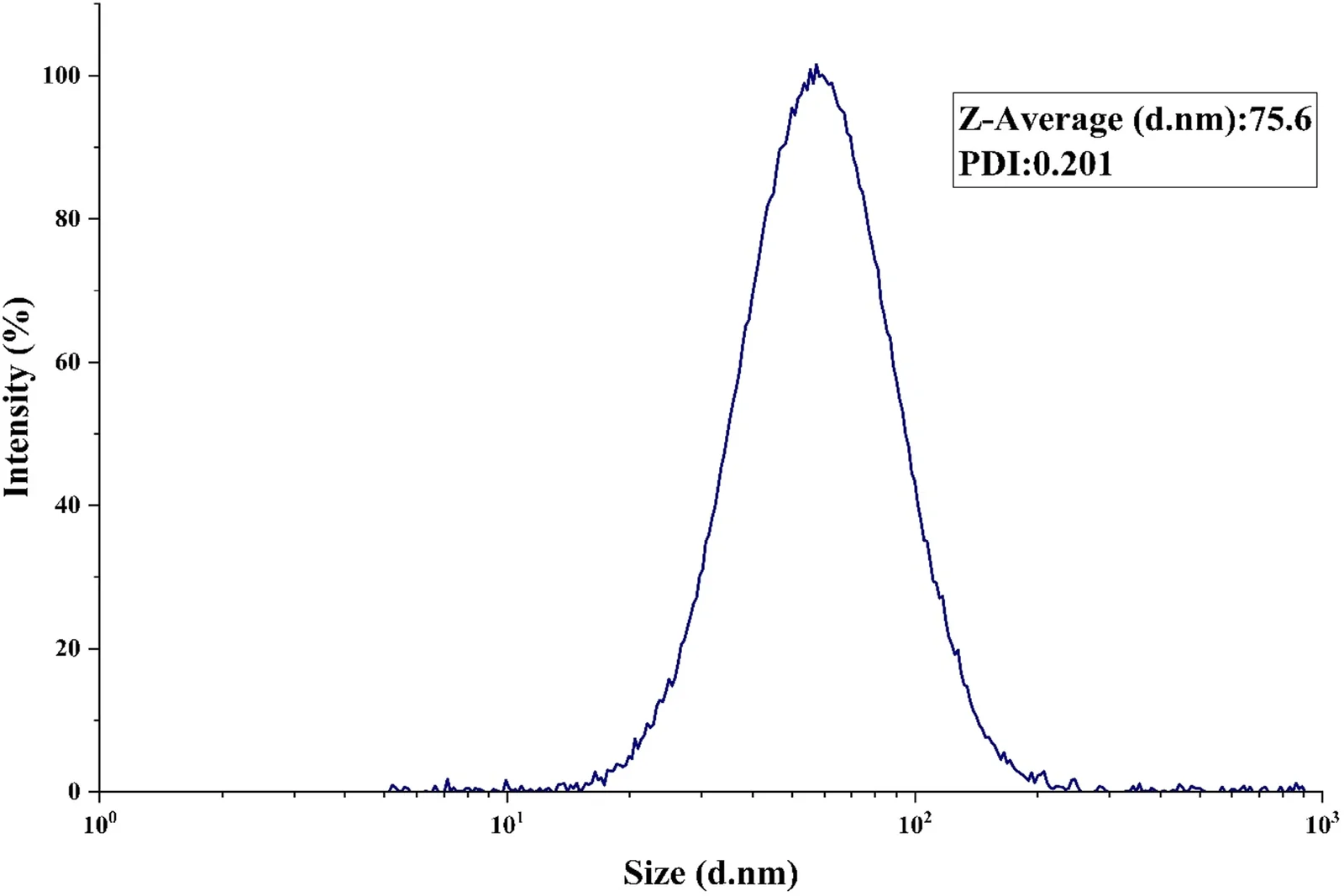
DLS intensity-weighted size distribution of biogenic *E. coli* EPS03-capped Ag/ZrO_2_ NCs showing a dominant population at 75.6 nm (PDI = 0.201; *Z*-average = 75.6 nm).

The biogenic Ag/ZrO_2_ NCs recorded a mean zeta potential of −33.54 mV, crossing the ±30 mV threshold that marks colloidal stability against particle aggregation (Fig. 9). The negative charge traces back to the deprotonated carboxyl and hydroxyl groups of the EPS corona, in line with the O–H and C=O bands recorded in FTIR. The distribution is unimodal from approximately −60 to −10 mV, with a single symmetric peak centred at the mean and no secondary population, reflecting a chemically uniform particle surface throughout the NC suspension.

**Fig. 9:**
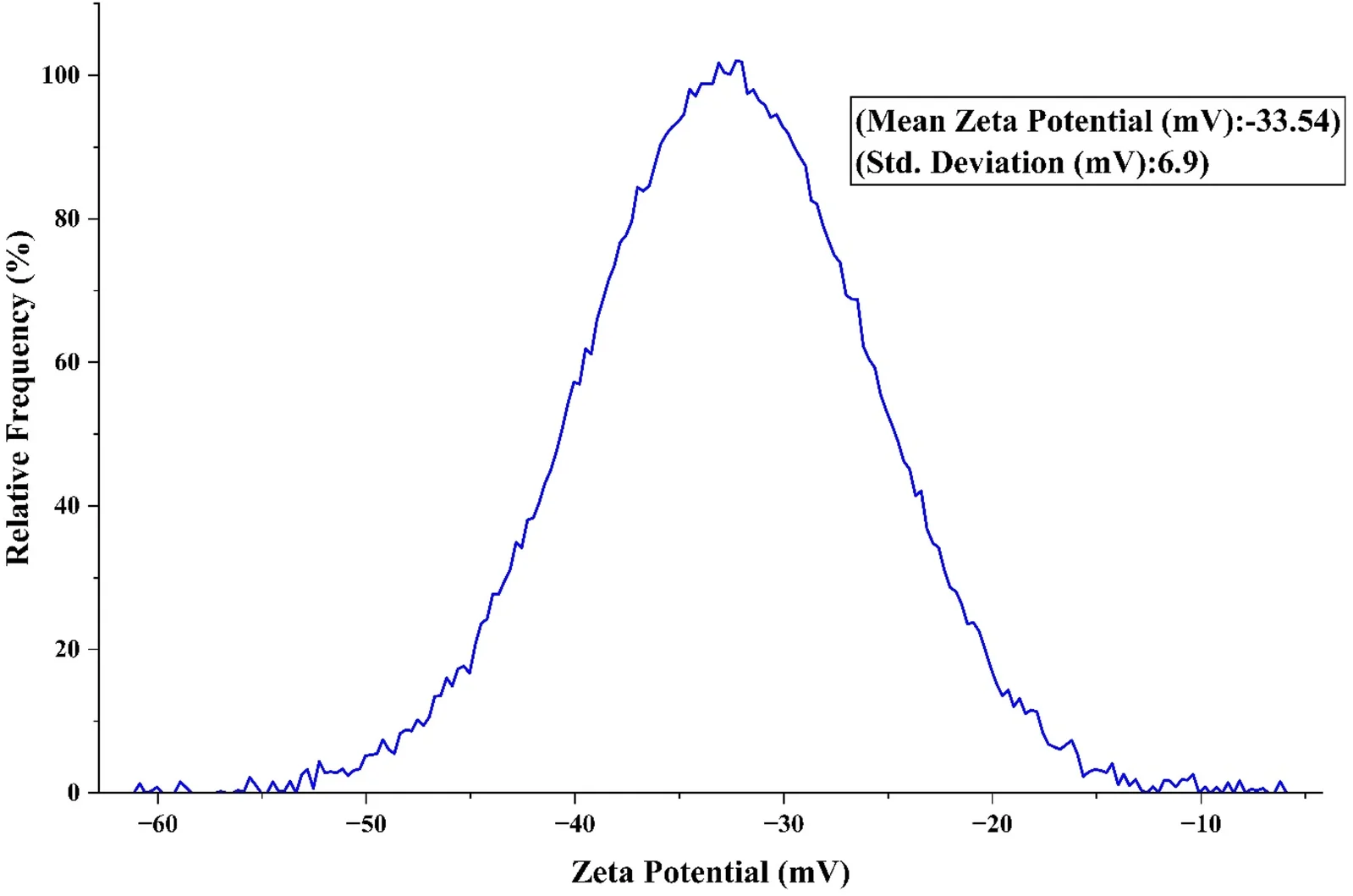
Zeta potential distribution of biogenic *E. coli* EPS03-capped Ag/ZrO_2_ NCs with a mean surface charge of −33.54 mV.

### Biomedical evaluation of Ag/ZrO_2_ NCs

#### Cytotoxicity of Ag/ZrO_2_ NCs against WI38

The MTT assay showed WI38 viability of 99.95%, 99.52%, and 95.51% at 31.25, 62.5, and 125 µg mL^−1^, falling to 51.11% at 250 µg mL^−1^ and further to 4.11% and 2.66% at 500 and 1000 µg mL^−1^, with an IC_50_ of 292.66 ± 1 µg mL^−1^ (Fig. 10). Up to 125 µg mL^−1^, viability remained above the 70% cutoff set by ISO 10993-5:2009, and the biogenic Ag/ZrO_2_ NCs meet this standard's criteria for biocompatibility across this concentration range. Cell morphology at 31.25–125 µg mL^−1^ matched that of the untreated control, with a normal spindle-shaped, confluent monolayer and no rounding, shrinkage, or detachment. In contrast, structural damage appeared only from 250 µg mL^−1^ onward, consistent with the drop below the 70% viability threshold.

**Fig. 10:**
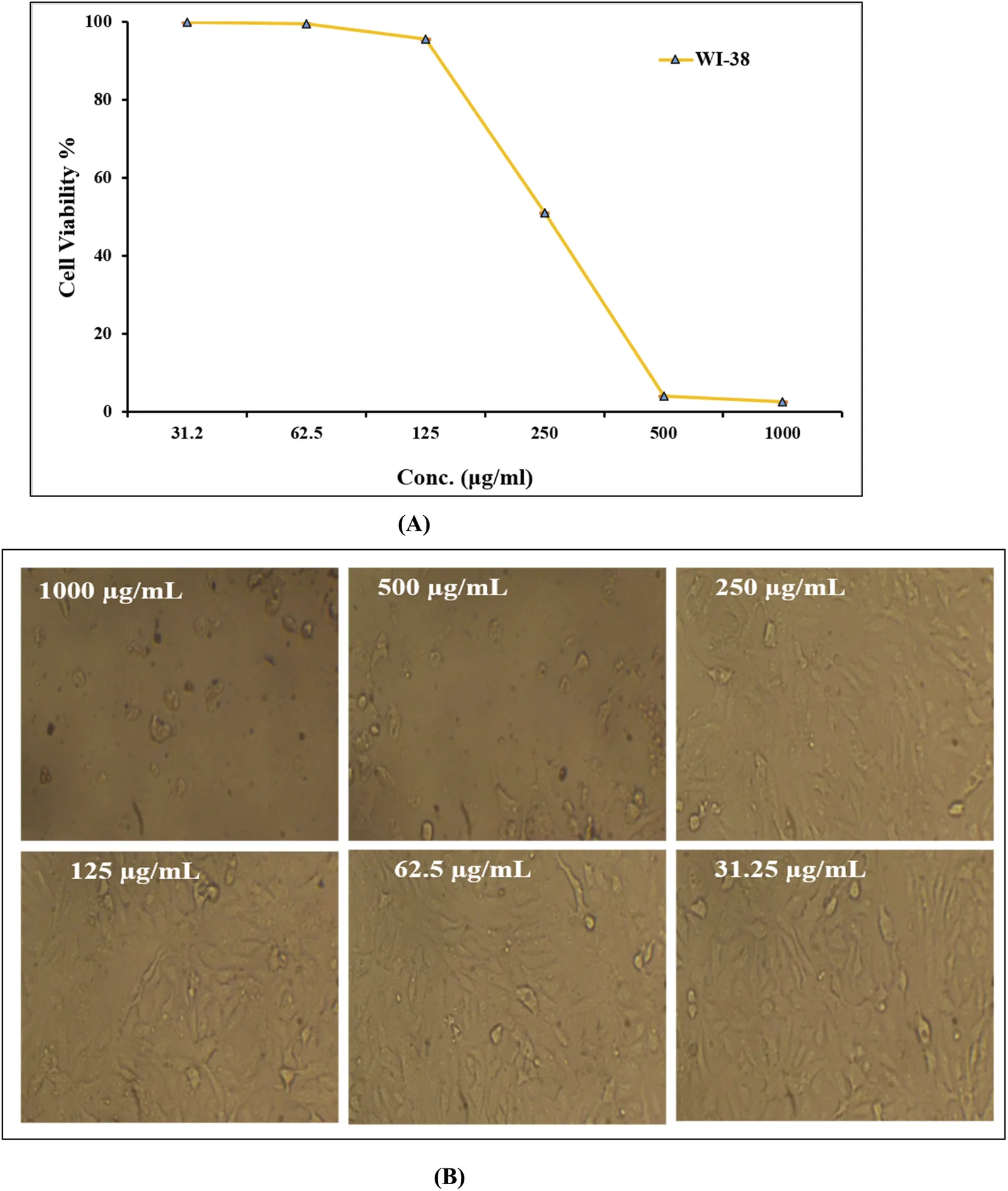
MTT assay of Ag/ZrO_2_ NCs on WI-38 fibroblasts. (A) Viability at 31.25–1000 µg mL^−1^ (IC_50_ = 292.66 ± 1 µg mL^−1^). (B) Cell morphology across the tested concentration range.

In comparison with NPs of physical or chemical origin, which generally recorded lower IC_50_ values against normal cells, ZrO_2_NPs were produced through the sol–gel route and tested against MRC-5 normal human lung fibroblasts, showing an IC_50_ value of 31.5 µg mL^−1^.^49^ Similarly, AgNPs were obtained by chemically reducing AgNO_3_ with trisodium citrate, and when applied to WI-38 cells, the IC_50_ dropped sharply to 2.18 µg mL^−1^.^50^ Meanwhile, silver zirconium phosphate was incorporated into a nano-based polymeric foam dressing through a precipitation approach, and testing on L929 mouse fibroblasts gave an IC_50_ of 3.8 µg mL^−1^.^51^ Also, AgNPs were physically fabricated *via* electrolysis by passing a constant current of 12 V through pure silver electrodes. When tested on NIH-3T3 fibroblasts, the IC_50_ was near 4 ppm (4 µg mL^−1^). Pushing the dose to 5 ppm left only about 5% of cells alive.^52^

#### Antioxidant testing by DPPH and ABTS^˙+^ tests

The antioxidant activity of biogenic Ag/ZrO_2_ NCs was assessed using the DPPH and ABTS radical-scavenging assays, with ascorbic acid as the reference standard. Both the NCs and ascorbic acid showed concentration-dependent activity across the tested range of 1.9–1000 µg mL^−1^. At 1.9 µg mL^−1^, the Ag/ZrO_2_ NCs scavenged 34.7% of DPPH radicals, while ascorbic acid reached 44.9%, a gap of 10.2 percentage points at the lowest tested dose. At 31.2 µg mL^−1^, the values were 66.6% and 73.7% respectively, reflecting a narrowing margin of 7.1 percentage points at mid-range concentrations. The IC_50_ values were 7.14 ± 0.13 µg mL^−1^ for the NCs and 2.69 ± 0.02 µg mL^−1^ for ascorbic acid. From 62.5 µg mL^−1^ onward, both samples tracked closely: 74.7% *vs.* 82.2% at 62.5 µg mL^−1^, and 89.5% *vs.* 92.7% at 250 µg mL^−1^ (Table 2). At the maximum tested concentration of 1000 µg mL^−1^, the NCs reached 96.8%, compared with 98.5% for ascorbic acid, a difference of only 1.7 percentage points, indicating near-convergent activity at saturation.

**Table 2:** DPPH and ABTS˙^+^ radical scavenging activity (%) of Ag/ZrO_2_ NCs and ascorbic acid at concentrations of 1.9–1000 µg mL^−1^, with corresponding IC_50_ values^a^

Conc. (µg mL^−1^)	Antioxidant scavenging activity			
	Ag/ZrO_2_ NCs	Ascorbic acid DPPH scavenging% IC_50_ = 2.69 ± 0.02 µg mL^−1^	Ag/ZrO_2_ NCs	Ascorbic acid
	DPPH scavenging%		ABTS˙^+^ scavenging%	ABTS˙^+^ scavenging%
	IC_50_ = 7.14 ± 0.13 µg mL^−1^		IC_50_ = 6.68 ± 0.12 µg mL^−1^	IC_50_ = 3.48 ± 0.10 µg mL^−1^
1.9	34.7***	44.9	37.3***	42.9
3.9	42.4***	52.5	43.1***	53.6
7.8	50.5***	58	52.4***	56.7
15.6	58.5***	66.2	59.0***	62.9
31.2	66.6***	73.7	65.1***	68.3
62.5	74.7***	82.2	71.1**	73.5
125	82.5***	89.7	80.8*	82.2
250	89.5***	92.7	87.0*	88.1
500	94.0***	96.1	91.8***	94.2
1000	96.8**	98.5	94.5**	96.1

a Values are means of three independent replicates (*n* = 3). *p < 0.05, **p < 0.01, ***p < 0.001, Ag/ZrO_2_ NCs *vs.* ascorbic acid at the matching concentration, independent-samples *t*-test

Moving to the ABTS˙^+^ test, at 1.9 µg mL^−1^, the NCs scavenged 37.3% of ABTS˙^+^ radicals, while ascorbic acid reached 42.9%, a gap of 5.6 percentage points, noticeably smaller than the corresponding DPPH gap at the same concentration. At 31.2 µg mL^−1^, the values were 65.1% and 68.3% respectively, a separation of just 3.2 percentage points, indicating that the NCs track the reference standard more closely in this assay across the mid-range. The IC_50_ values were 6.68 ± 0.12 µg mL^−1^ for the NCs and 3.48 ± 0.10 µg mL^−1^ for ascorbic acid (Table 2). At 62.5 µg mL^−1^, scavenging was 71.1% *vs.* 73.5%, and by 250 µg mL^−1^, the NCs reached 87.0% *vs.* 88.1% for ascorbic acid, a margin of just 1.1 percentage points. At 1000 µg mL^−1^, the NCs recorded 94.5%, compared with 96.1% for ascorbic acid, indicating nearequivalent activity at the upper end of the tested range.

Following our results, the marine macroalga *Sargassum tenerrimum*-mediated spherically agglomerated Ag/ZrO_2_ NCs represent the sole documented biogenic route, exhibiting dose-dependent DPPH scavenging across 10–50 µg mL^−1^.^53^*Fusarium oxysporum* yielded extracellular nanocrystalline ZrO_2_*via* protein-mediated hydrolysis at ambient temperature.^54^ For the Ag NP fraction, *Trichoderma atroviride*-mediated anisotropic NPs of 15–25 nm recorded a DPPH IC_50_ of 45.6 µg mL^−1^,^55^ while *Trichoderma saturnisporum*-mycosynthesized spherical NPs of 10–70 nm yielded a DPPH IC_50_ of 73.5 µg mL^−1^.^56^ More potently, *Streptomyces violaceus* exopolysaccharide-capped Ag NPs achieved 89.5% DPPH inhibition at 50 µg mL^−1^, surpassing ascorbic acid (49.6%) at the same concentration, an effect attributed to the electron-donating capacity of microbial capping agents on the nanoparticle surface.^57^

The concentration-dependent radical scavenging observed in both assays can be traced to three contributing sources. The EPS corona hydroxyl and carboxyl groups, confirmed by O–H stretching at 3444.81 cm^−1^ and C=O stretching at 1636 cm^−1^, quench free radicals through hydrogen atom transfer and single-electron transfer mechanisms.^58^ The sub-30 nm Ag domains dispersed across the ZrO_2_/EPS matrix amplify the surface electron-donating capacity through their high surface-to-volume ratio.^59^ Surface Zr–OH groups at 874 cm^−1^ contribute further through redox cycling and ROS neutralization.^60^ The closer IC_50_ alignment with ascorbic acid in ABTS relative to DPPH reflects ABTS's greater sensitivity to single-electron transfer, a pathway that the EPS-functionalized surface facilitates more readily.

### Anti-inflammatory evaluation of Ag/ZrO_2_ NCs

Ag/ZrO_2_ NCs inhibited COX-1 concentration-dependently across 0.5–1000 µg mL^−1^, recording 18.3% at 0.5 µg mL^−1^, 39.2% at 2 µg mL^−1^, and 51.2% at 7.8 µg mL^−1^. Inhibition continued to rise, reaching 63.2% at 31.25 µg mL^−1^, 78.2% at 125 µg mL^−1^, and 86.9% at 250 µg mL^−1^, and then 97.8% at 1000 µg mL^−1^ (Fig. 11). The IC_50_ was 7.43 ± 0.2 µg mL^−1^, approximately 2.8-fold higher than that of celecoxib (IC_50_ = 2.66 ± 0.3 µg mL^−1^), yet indicative of potent suppressive activity at low concentrations.

**Fig. 11:**
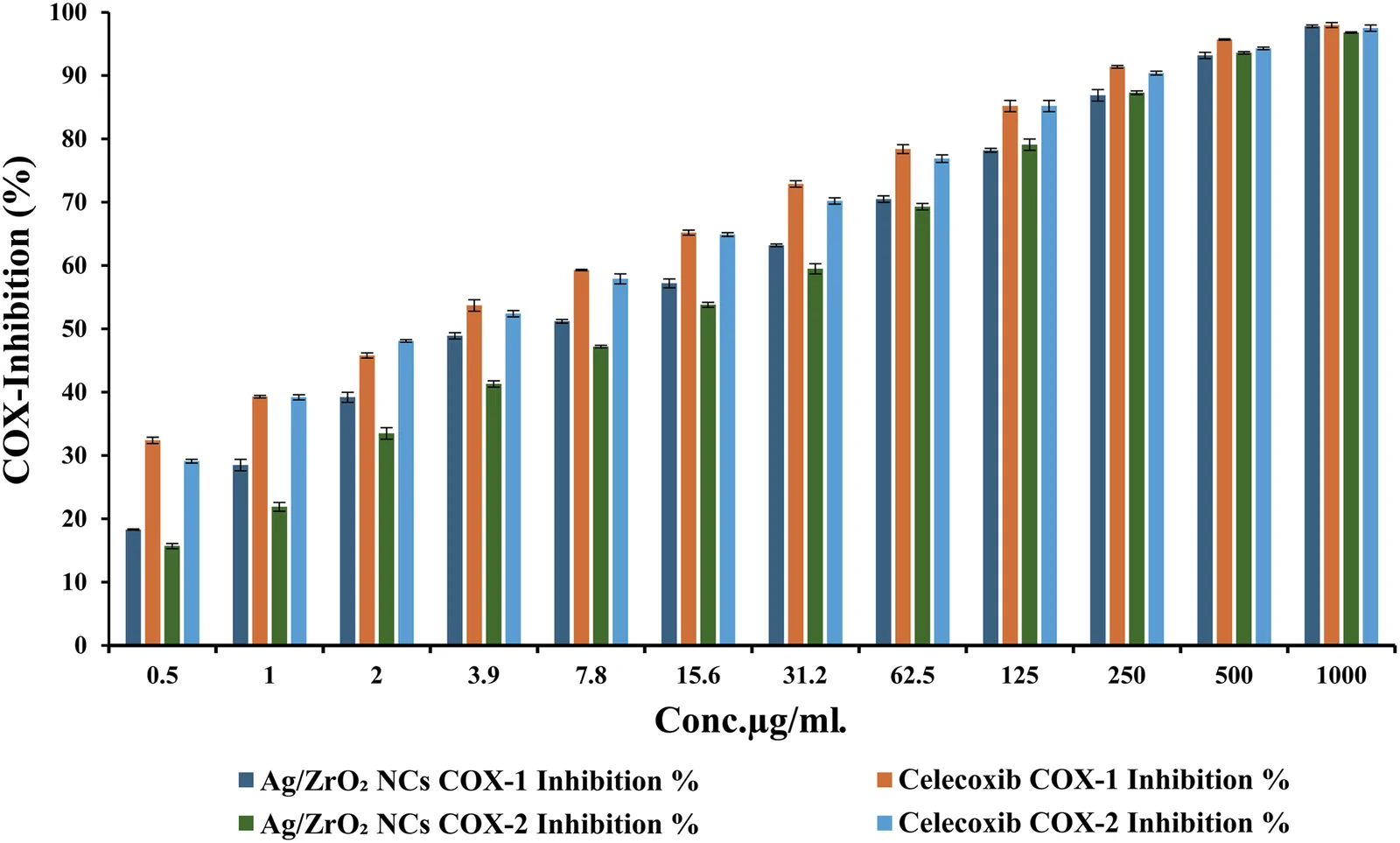
COX-1 and COX-2 inhibition (%) by Ag/ZrO_2_ NCs and celecoxib across a concentration range of 0.5–1000 µg mL^−1^. Bars represent mean ± SD, *n* = 3 independent replicates.

Against COX-2, Ag/ZrO_2_ NCs recorded 15.7% inhibition at 0.5 µg mL^−1^, 33.5% at 2 µg mL^−1^, and 47.2% at 7.8 µg mL^−1^, crossing the 50% threshold between 7.8 and 15.6 µg mL^−1^, where inhibition reached 53.8%. At higher concentrations, inhibition stood at 69.3% at 62.5 µg mL^−1^, 87.3% at 250 µg mL^−1^, and 96.8% at 1000 µg mL^−1^ (Fig. 11). The IC_50_ was 10.5 ± 0.1 µg mL^−1^, roughly 3.2-fold above celecoxib (IC_50_ = 3.27 ± 0.3 µg mL^−1^). The COX-1 IC_50_ of 7.43 µg mL^−1^ was lower than the COX-2 IC_50_ of 10.5 µg mL^−1^, indicating marginally greater selectivity toward COX-1, a pattern worth noting given that preferential COX-2 inhibition is generally associated with a reduced gastrointestinal side-effect profile.

Concerning the BSA test, Ag/ZrO_2_ NCs inhibited heat-induced BSA denaturation across the full tested range of 1.5–200 µg mL^−1^ in a concentration-dependent pattern. At the lowest concentration of 1.5 µg mL^−1^, inhibition reached 42.3%, rising through 50.8% at 3 µg mL^−1^ and 63.9% at 6.25 µg mL^−1^. At mid-range concentrations, inhibition was 68.2% at 12.5 µg mL^−1^ and 76.4% at 25 µg mL^−1^, then increased to 83.3% at 50 µg mL^−1^ and 90.0% at 100 µg mL^−1^, reaching a maximum of 96.7% at 200 µg mL^−1^ (Fig. 12). The IC_50_ was 2.46 ± 0.112 µg mL^−1^, compared to diclofenac sodium (IC_50_ = 1.72 ± 0.022 µg mL^−1^), representing a 1.4-fold difference. This narrow margin places Ag/ZrO_2_ NCs in proximity to the reference drug in potency for denaturation suppression.

**Fig. 12:**
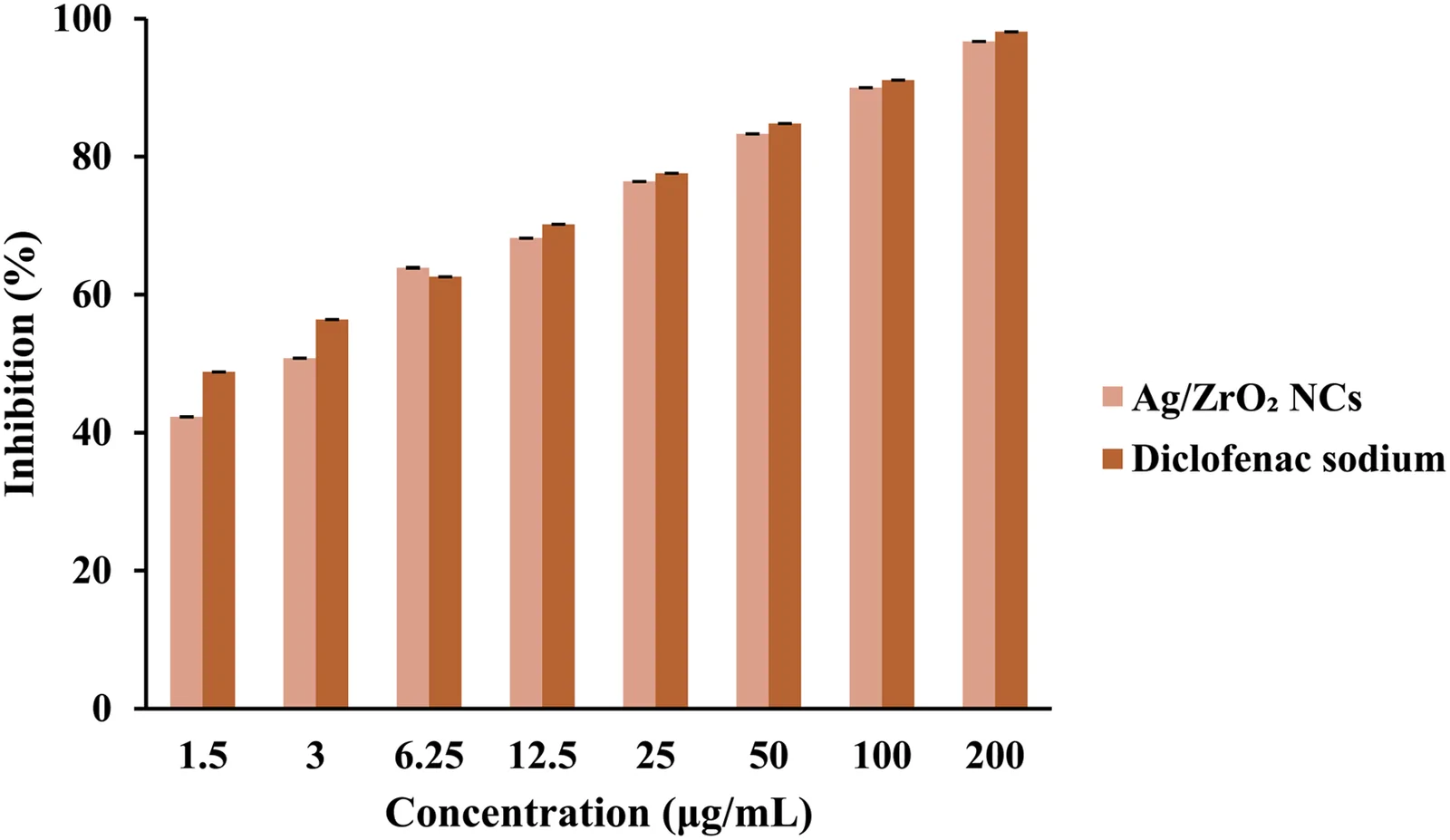
BSA denaturation inhibition (%) by Ag/ZrO_2_ NCs and diclofenac sodium across a concentration range of 1.5–200 µg mL^−1^. Data represented as mean ± SD, *n* = 3.

In accordance with our findings, *Saussurea costus* leaf and root extract-derived AgNPs recorded over 99% inhibition of COX-1, 5-LOX, and secreted PLA_2_ enzymes at 120 µg mL^−1^.^61^*Lantana montevidensis* leaf extract-mediated spherical AgNPs of 98.64 nm suppressed carrageenan-induced paw edema by 92.95% at 6 h, with anti-arthritic activity reaching 97.26%, alongside marked drops in TNF-α (62.91 ± 0.01 pg mL^−1^) and IL-6 (136 ± 3 pg mL^−1^) levels.^62^

Turning to ZrO_2_ NPs, clove and cardamom extract-reinforced spherical ZrO_2_ NPs of 5–20 nm showed dose-dependent anti-inflammatory activity *via* BSA denaturation across concentrations of 10–50 µL.^63^ Beyond that, propolis extract-mediated ZrO_2_ NPs, when formulated as ciprofloxacin-loaded chitosan nanocapsules, reached 94% anti-inflammatory inhibition.^64^ Also, *Rhododendron arboreum*-mediated ZrO_2_ NPs inhibited heat-induced albumin denaturation across 6.75–100 µg mL^−1^, with inhibition values spanning 60–97% in a concentration-dependent pattern.^65^

The anti-inflammatory activity of the Ag/ZrO_2_ NCs may be attributed to three contributing factors: released Ag^+^ ions, ZrO_2_ surface chemistry, and the EPS corona. The COX-1 and COX-2 IC_50_ values of 7.43 ± 0.2 and 10.5 ± 0.1 µg mL^−1^ reflect sustained Ag^+^ release from the ZrO_2_ scaffold. For BSA denaturation, the IC_50_ values of 2.46 ± 0.112 µg mL^−1^ and 1.72 ± 0.022 µg mL^−1^ for diclofenac sodium reflect protein-stabilising activity at the NC surface. Zr–OH groups on the 15.5 nm monoclinic ZrO_2_ crystallites form hydrogen bonds with polar BSA residues under heat stress, limiting unfolding. The EPS amide groups at 1507–1557 cm^−1^ provide further stabilisation through hydrogen bonding with BSA backbone linkages, consistent with findings for polysaccharide-coated nanoparticles.^66^ The narrow 1.4-fold difference between the NCs and diclofenac sodium is worth noting within the tested concentration range.

### Antimicrobial screening of Ag/ZrO_2_ NCs

Against Gram-positive organisms, Ag/ZrO_2_ NCs performed strongest against *Bacillus subtilis* (34 *vs.* 29 for gentamicin), followed by *S. aureus* (26 *vs.* 24), with both exceeding the reference drug. Against MRSA, the nanocomposite (18) again surpassed gentamicin (14), which carries practical weight given MRSA's notorious resistance profile. The only reversal was in *Enterococcus faecalis*, where gentamicin (20) slightly edged out the nanocomposite (19) (Fig. 13A).

**Fig. 13:**
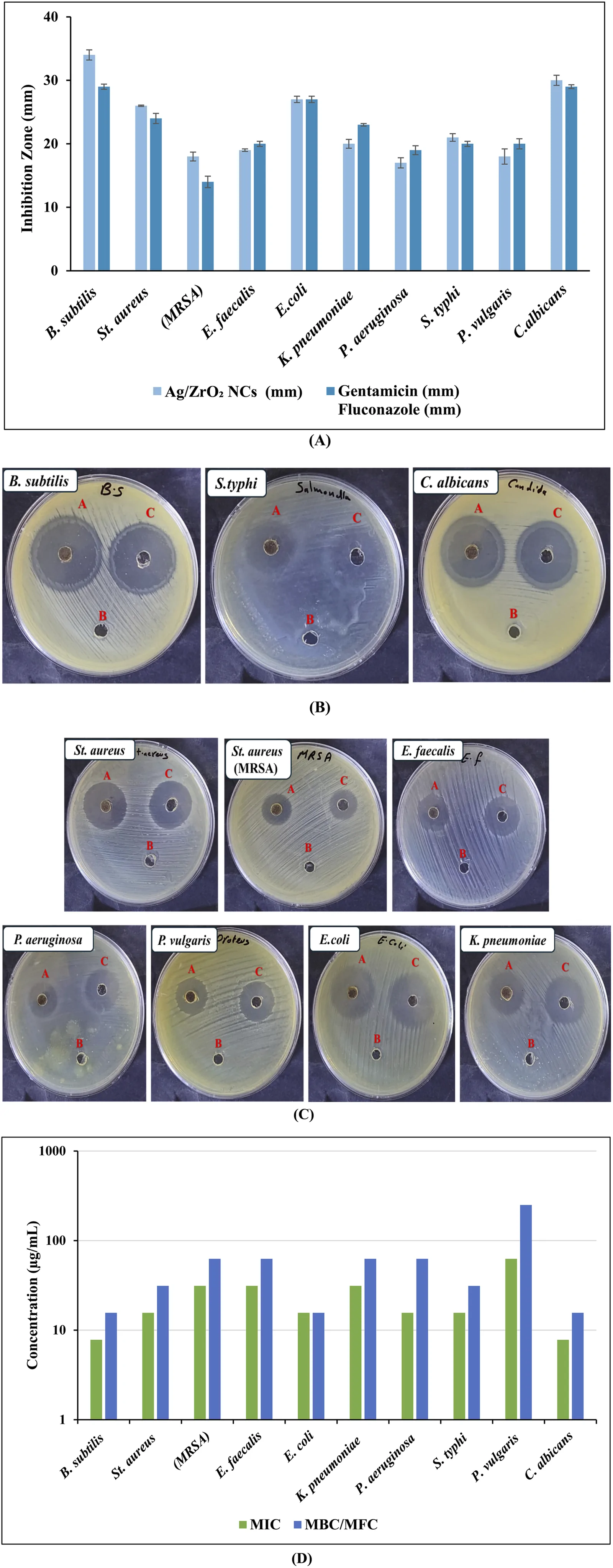
(A) Inhibition zones (mm) of Ag/ZrO_2_ NCs *vs.* gentamicin across all tested microbial strains. (B and C) Agar well diffusion plates illustrating inhibition zones produced by the Ag/ZrO_2_ NCs against tested strains; A = Ag/ZrO_2_ NCs, B = negative control, C = gentamicin (bacteria)/fluconazole (fungi). (D) MIC and MBC/MFC (µg mL^−1^) of Ag/ZrO_2_ NCs against tested microbial strains. The *y*-axis is presented on a logarithmic scale.

Among Gram-negative bacteria, *E. coli* showed equal susceptibility to both (27). In contrast, gentamicin outperformed the nanocomposite against *K. pneumoniae* (23 *vs.* 20), *P. aeruginosa* (19 *vs.* 17), and *P. vulgaris* (20 *vs.* 18), likely reflecting the outer membrane barrier that limits nanoparticle penetration in these organisms(Fig. 13C). Conversely, the nanocomposite held a marginal advantage over gentamicin against *S. typhi* (21 *vs.* 20). Against *Candida albicans*, Ag/ZrO_2_ NCs (30) closely matched fluconazole (29), a result worth considering given the increasing prevalence of fluconazole-resistant strains (Fig. 13B).

The broth microdilution method extended the zone-based screening by quantifying MIC, MBC, and MFC for the Ag/ZrO_2_ NCs across the same panel of reference strains. Among the G + ve isolates, the MIC was lowest against *B. subtilis* at 7.8 µg mL^−1^, followed by *S. aureus* at 15.62 µg mL^−1^, while MRSA and *E. faecalis* each required 31.25 µg mL^−1^ for growth inhibition. G-ve strains showed a comparable spread, with *E. coli*, *P. aeruginosa*, and *S. typhi* inhibited at 15.62 µg mL^−1^, *K. pneumoniae* at 31.25 µg mL^−1^, and *P. vulgaris* requiring the highest concentration in the panel at 62.5 µg mL^−1^(Fig. 13D).

MBC values followed the same ranking: 15.62 µg mL^−1^ for *B. subtilis*, 31.25 µg mL^−1^ for *S. aureus*, and 62.5 µg mL^−1^ for both MRSA and *E. faecalis*, giving MBC/MIC ratios of 2 across all four G + ve strains, within the bactericidal cutoff (≤4) for each. Among G-ve strains, *E. coli* carried the tightest ratio at 1 (MBC 15.62 µg mL^−1^), *S. typhi* and *K. pneumoniae* reached ratios of 2 (MBC 31.25 and 62.5 µg mL^−1^, respectively), while *P. aeruginosa* and *P. vulgaris* both reached a ratio of 4 (MBC 62.5 and 250 µg mL^−1^, respectively), the upper edge of the bactericidal cutoff, and the weakest cidal performance in the panel. Against *C. albicans*, an MIC of 7.8 µg mL^−1^ and an MFC of 15.62 µg mL^−1^ yielded an MFC/MIC ratio of 2, within the fungicidal range (≤4) (Fig. 13D). The lowest ratios, and so the strongest bactericidal or fungicidal potency, belonged to *E. coli* (1), then *B. subtilis*, *S. aureus*, *S. typhi*, and *C. albicans* (2 each); *P. aeruginosa* and *P. vulgaris*, at a ratio of 4, also carried the smallest inhibition zones among their respective groups.

Following our results, ZrO_2_–Ag_2_O NCs ranging from 14 to 42 nm outperformed pure ZrO_2_ and Ag_2_O individually in disc diffusion assays against Gram-positive (*B. subtilis*, *S. mutans*, *S. aureus*) and Gram-negative (*E. coli*, *P. aeruginosa*, *K. oxytoca*) bacteria, with the *Z*-A2.0 formulation yielding the widest inhibition zones across all tested strains.^67^ Polyaniline/ZrO_2_–Ag nanohybrid of rod and spherical morphology (47–91 nm, monoclinic zirconia phase) equally showed substantial *in vitro* antibacterial activity.^68^ Biogenically synthesized ZrO_2_ NPs fabricated from *Passiflora edulis* produced inhibition zones of 8 mm against *S. aureus* at low concentrations and 11 mm against *S. mutans* at higher doses. In contrast, growth suppression of *S. aureus* and *K. pneumoniae* reached 100% at concentrations of 5–25%69. Similarly, ZrO_2_ NPs prepared from *Wrightia tinctoria* leaf extract produced measurable inhibition zones against both Gram-positive and Gram-negative strains at 10 µg mL^−1^, with phytochemical constituents credited for reducing particle size and expanding surface area.^70^

ZrO_2_ NPs (20–40 nm) embedded in 3D-printed dental resin substantially reduced *C. albicans* metabolic activity, with SEM confirming suppressed biofilm viability after three months of aging through ROS-mediated disruption of fungal hyphae.^71^ Spherical biogenic AgNPs (11–15 nm) from *Anabaena variabilis* showed antifungal activity against *C. albicans* planktonic cells and acted synergistically with fluconazole, with SEM revealing cell wrinkling, nuclear irregularities, and membrane rupture as the underlying damage mechanisms.^72^

The antimicrobial activity recorded for the Ag/ZrO_2_ NCs may be attributed to multiple interrelated physicochemical properties of the nanocomposite. The zeta potential of −33.54 mV reflects deprotonated carboxyl and hydroxyl groups within the EPS corona, confirmed by FTIR O–H (3444 cm^−1^) and C=O (1636 cm^−1^) absorptions. It is sufficient to maintain colloidal stability and particle bioavailability at the microbial surface. Nanoparticle contact with the bacterial envelope is mediated by van der Waals forces and localized charge heterogeneities, meaning the negative surface charge does not impede membrane interaction.^73^ XRD-derived Ag crystallite size (29.0 nm) places these domains under 30 nm, and TEM shows them as electron-dense particles distributed across the ZrO_2_/EPS matrix, a dispersion state consistent with the suppressed SPR in UV-vis, which maximizes surface area and supports sustained Ag^+^ release. Those released Ag^+^ ions penetrate the cell envelope, inactivate thiol-dependent respiratory enzymes, and induce intracellular ROS accumulation that degrades membrane lipids, proteins, and DNA.^74^ The 15.5 nm monoclinic ZrO_2_ crystallites identified by XRD against JCPDS 37-1484 carry surface Zr–OH groups confirmed by FTIR at 874 cm^−1^, contributing additional ROS generation through surface redox reactions and extending oxidative damage beyond what Ag^+^ alone produces.^75^ The EPS corona, identified by amide I/II (1507–1636 cm^−1^) and C–O–C (1015–1113 cm^−1^) FTIR bands, suppresses interparticle aggregation and preserves dispersion, maintaining particle bioavailability throughout the assay.^76^

The G+ve strains showed greater susceptibility to the Ag/ZrO_2_ NCs than their G-ve counterparts. Against *B. subtilis*, *S. aureus*, and MRSA, the NCs produced inhibition zones of 34, 26, and 18 mm, respectively, each exceeding those of gentamicin (29, 24, and 14 mm). Gram-negative species responded less strongly, with zones of 20, 17, and 18 mm recorded against *K. pneumoniae*, *P. aeruginosa*, and *P. vulgaris*. This outcome is explained by the lipopolysaccharide-rich outer membrane present in Gram-negative bacteria, which physically limits nanoparticle access to the inner membrane and reduces Ag^+^ influx into the cytoplasm.^77^ Gram-positive organisms lack this outer membrane layer, so the peptidoglycan wall poses considerably less resistance to particle contact and ion penetration.^78^ Against *C. albicans*, a 30 mm zone was recorded, marginally surpassing the fluconazole zone of 29 mm. Ag^+^ ions are known to interfere with ergosterol biosynthesis and disrupt fungal cell wall glucan assembly, which accounts for the antifungal potency observed here.^78^

These findings are confined to *in vitro* conditions (Table 3), and future studies should conduct *in vivo* experiments to isolate the individual contributions of each component to the observed biological activities. The formulation of Ag/ZrO_2_ NCs into delivery systems suitable for targeted therapeutic use also merits further investigation.

**Table 3:** Antioxidant, anti-inflammatory, and antimicrobial activities of the biogenic Ag/ZrO_2_ NCs relative to previously reported biogenic nanomaterials

Biogenic source	Nanomaterial	Reported activity
**Antioxidant activity**		
*E. coli* EPS03 (this study)	Ag/ZrO_2_ NCs	DPPH IC_50_ = 7.14 ± 0.13 µg mL^−1^ ABTS˙^+^ IC_50_ = 6.68 ± 0.12 µg mL^−1^
*Sargassum tenerrimum*	Ag/ZrO_2_ NCs, spherically agglomerated	Dose-dependent DPPH scavenging, 10–50 µg mL^−1^ (ref. 49)
*Trichoderma atroviride*	AgNPs, 15–25 nm, anisotropic	DPPH IC_50_ = 45.6 µg mL^−1^ (ref. 53)
*Trichoderma saturnisporum*	AgNPs, 10–70 nm, spherical	DPPH IC_50_ = 73.5 µg mL^−1^ (ref. 56)
*Streptomyces violaceus* (EPS-capped)	AgNPs	89.5% DPPH inhibition at 50 µg mL^−1^ (ref. 57)

**Anti-inflammatory activity**		
*E. coli* EPS03 (this study)	Ag/ZrO_2_ NCs	COX-1 IC_50_ = 7.43 µg mL; COX-2 IC_50_ = 10.5 µg mL; BSA IC_50_ = 2.46 µg mL^−1^
*Saussurea costus* (leaf/root extract)	AgNPs	>99% inhibition of COX-1, 5-LOX and secreted PLA2 at 120 µg mL^−1^ (ref. 61)
*Lantana montevidensis*	AgNPs, 98.64 nm, spherical	92.95% paw-oedema suppression at 6 h; TNF-α 62.91 pg mL^−1^, IL-6136 pg mL^−1^ (ref. 62)
Clove and cardamom extract	ZrO_2_ NPs, 5–20 nm, spherical	Dose-dependent BSA denaturation inhibition, 10–50 µL (ref. 63)
Propolis extract	ZrO_2_ NPs (ciprofloxacin-loaded chitosan nanocapsules)	94% anti-inflammatory inhibition^64^
*Rhododendron arboreum*	ZrO_2_ NPs	BSA denaturation inhibition 60–97%, 6.75–100 µg mL^−1^ (ref. 65)

**Antimicrobial activity**		
*E. coli* EPS03 (this study)	Ag/ZrO_2_ NCs	Inhibition zones 17–34 mm *vs.* bacteria; 30 mm *vs. C. albicans*
*Passiflora edulis*	ZrO_2_ NPs	8 mm *vs. S. aureus*; 11 mm *vs. S. mutans*; 100% growth suppression at 5–25% (ref. 69)
*Wrightia tinctoria* (leaf extract)	ZrO_2_ NPs	Measurable inhibition zones *vs.* Gram-positive/-negative strains at 10 µg mL^−1^ (ref. 70)
*Anabaena variabilis*	AgNPs, 11–15 nm, spherical	Antifungal activity *vs. C. albicans*; synergistic with fluconazole^72^

## Conclusion

This study reports the biogenic synthesis of Ag/ZrO_2_ NCs using *E. coli* EPS03 as a dual reducing and capping agent. Physicochemical characterization confirmed the formation of a well-defined nanocomposite, with monoclinic ZrO_2_ and FCC Ag occupying separate crystalline domains, Ag finely dispersed across the ZrO_2_/EPS matrix, and the EPS corona retained at the particle surface. The negative surface charge was sufficient to maintain colloidal stability throughout the suspension. Biomedical evaluation demonstrated concentration-dependent antioxidant activity in both DPPH and ABTS assays, with IC_50_ values approaching those of ascorbic acid. Anti-inflammatory testing showed meaningful inhibition of COX-1 and COX-2, along with potent suppression of BSA denaturation. Antimicrobial screening revealed broad-spectrum activity, with the NCs surpassing gentamicin against several Gram-positive strains, including MRSA, matching it against *E. coli*, and closely approaching fluconazole against *Candida albicans*.

The recorded activities across these three pathways trace back to a shared physicochemical basis: sustained Ag^+^ release from sub-nanometer dispersed domains, surface redox contributions from monoclinic ZrO_2_ crystallites, and the biological stabilizing role of the EPS corona. Taken together, these results support the Ag/ZrO_2_ NC system as a biogenic multifunctional platform warranting further evaluation in cytotoxicity and *in vivo* models.

## Author contributions

Methodology: I. M. I, Z. F. Z, H. A. A, E. M. K, M. O. I. A, D. A, F. M. K. A, investigation: M. K. A, N. A, A. E. A., D. A, A. G, visualization and data curation: N. A, M. K. A, F. M. K. A, D. A, formal analysis: N. A, D. A, A. G, Z. F. A, H. A. A, writing – review and editing: A. G.

## Conflicts of interest

None to be declared.

## Data Availability

The data supporting this study are available from the corresponding author upon reasonable request.
